# SIVcol Nef counteracts SERINC5 by promoting its proteasomal degradation but does not efficiently enhance HIV-1 replication in human CD4+ T cells and lymphoid tissue

**DOI:** 10.1371/journal.ppat.1007269

**Published:** 2018-08-20

**Authors:** Dorota Kmiec, Bengisu Akbil, Swetha Ananth, Dominik Hotter, Konstantin M. J. Sparrer, Christina M. Stürzel, Birthe Trautz, Ahidjo Ayouba, Martine Peeters, Zhong Yao, Igor Stagljar, Vânia Passos, Thomas Zillinger, Christine Goffinet, Daniel Sauter, Oliver T. Fackler, Frank Kirchhoff

**Affiliations:** 1 Institute of Molecular Virology, Ulm University Medical Center, Ulm, Germany; 2 Department of Infectious Diseases, Integrative Virology, CIID, University Hospital Heidelberg, Heidelberg, Germany; 3 German Center for Infection Research (DZIF), Partner Site Heidelberg, Heidelberg, Germany; 4 TransVIHMI, Institut de Recherche pour le Développement, University of Montpellier, INSERM, Montpellier, France; 5 Donnelly Centre, University of Toronto, Ontario, Canada; 6 Departments of Biochemistry and Molecular Genetics, University of Toronto, Ontario, Canada; 7 Institute of Virology, Hannover Medical School, Hannover, Germany; 8 Institute of Clinical Chemistry and Clinical Pharmacology, University of Bonn, Bonn, Germany; Fred Hutchinson Cancer Research Center, UNITED STATES

## Abstract

SERINC5 is a host restriction factor that impairs infectivity of HIV-1 and other primate lentiviruses and is counteracted by the viral accessory protein Nef. However, the importance of SERINC5 antagonism for viral replication and cytopathicity remained unclear. Here, we show that the Nef protein of the highly divergent SIVcol lineage infecting mantled guerezas (*Colobus guereza*) is a potent antagonist of SERINC5, although it lacks the CD4, CD3 and CD28 down-modulation activities exerted by other primate lentiviral Nefs. In addition, SIVcol Nefs decrease CXCR4 cell surface expression, suppress TCR-induced actin remodeling, and counteract *Colobus* but not human tetherin. Unlike HIV-1 Nef proteins, SIVcol Nef induces efficient proteasomal degradation of SERINC5 and counteracts orthologs from highly divergent vertebrate species, such as *Xenopus* frogs and zebrafish. A single Y86F mutation disrupts SERINC5 and tetherin antagonism but not CXCR4 down-modulation by SIVcol Nef, while mutation of a C-proximal di-leucine motif has the opposite effect. Unexpectedly, the Y86F change in SIVcol Nef had little if any effect on viral replication and CD4+ T cell depletion in preactivated human CD4+ T cells and in *ex vivo* infected lymphoid tissue. However, SIVcol Nef increased virion infectivity up to 10-fold and moderately increased viral replication in resting peripheral blood mononuclear cells (PBMCs) that were first infected with HIV-1 and activated three or six days later. In conclusion, SIVcol Nef lacks several activities that are conserved in other primate lentiviruses and utilizes a distinct proteasome-dependent mechanism to counteract SERINC5. Our finding that evolutionarily distinct SIVcol Nefs show potent anti-SERINC5 activity supports a relevant role of SERINC5 antagonism for viral fitness *in vivo*. Our results further suggest this Nef function is particularly important for virion infectivity under conditions of limited CD4+ T cell activation.

## Author summary

The accessory protein Nef promotes primate lentiviral replication and enhances the pathogenicity of HIV-1 by mechanisms of immune evasion and enhancing viral infectivity and replication. Here, we show that the evolutionarily most isolated primate lentivirus SIVcol lacks several otherwise conserved Nef functions. Nevertheless, SIVcol Nef potently antagonizes SERINC5, a recently discovered inhibitor of viral infectivity, by down-modulating it from the cell surface and inducing its proteasomal degradation. We identified Y86 in SIVcol Nef as a key determinant of SERINC5 antagonism. Efficient counteraction of SERINC5 did not increase HIV-1 replication in preactivated CD4+ T cells and in *ex vivo* infected lymphoid tissue but had modest enhancing effects when resting PBMCs were first infected and activated six days later. Evolution of high anti-SERINC5 activity by SIVcol Nef supports a relevant role of this antagonism *in vivo*, for instance by enhancing virion infectivity under conditions of limited T cell activation.

## Introduction

The accessory *nef* gene is present in the genomes of all primate lentiviruses that infect at least forty different African monkey species as well as great apes and humans. Nef performs a striking number of activities [1,2] and is required for efficient viral replication and pathogenicity of HIV-1 and SIVmac in humans and experimentally infected rhesus macaques, respectively [3–5]. Some Nef functions are conserved in the vast majority of primate lentiviruses. These include down-modulation of the CD4 receptor and class I major histocompatibility complex (MHC-I) from the cell surface [6], enhancement of virion infectivity [7] by counteraction of the antiviral factor SERINC5 [8–10], modulation of the actin skeleton [11,12] and T cell signaling and migration [13,14], as well as stimulation of NF-κB activity. The Nef proteins of HIV-2, which originated from several cross-species transmissions of SIVsmm found in sooty mangabeys, and most SIVs additionally down-modulate CD3 from the cell surface to suppress stimulation of virally infected CD4+ T cells and antiviral gene expression [6,15–17]. In contrast, this Nef function was lost in most primate lentiviruses encoding a *vpu* gene, *i*.*e*. HIV-1, its direct simian precursors SIVcpz and SIVgor from chimpanzees and gorillas, respectively, and some closely related SIVs infecting several *Cercopithecus* species [6,18]. These primate lentiviruses are unable to block TCR-CD3-mediated T cell activation and instead use Vpu to suppress antiviral gene expression by inhibiting activation of the transcription factor NF-κB [19,20]. Most primate lentiviruses lacking Vpu as well as SIVcpz, SIVgor and HIV-1 group O also use Nef to antagonize the restriction factor tetherin to allow efficient release of viral particles from infected cells [21–24]. Finally, many HIV-2, SIV and (to a lesser extent) HIV-1 Nef proteins down-modulate CD28 and CXCR4 from the cell surface [14,25,26]. Thus, the multifunctionality of lentiviral Nefs highlights the importance of this accessory protein but also poses a challenge for dissecting its effects on viral replication and pathogenicity.

Previous studies suggested that the Nef protein of SIVcol infecting mantled guerezas (*Colobus guereza*) is functionally distinct from all other primate lentiviral Nefs. SIVcol is highly divergent from other primate lentiviruses with average amino acid identities of only 40 to 50% for Gag and Pol and about 25% for Nef [27]. To our current knowledge, SIVcol Nef proteins show little if any activity in a variety of functions that are otherwise conserved, *i*.*e*. down-modulation of CD4, CD28 and CD3 [20,28]. However, SIVcol Nef is highly active in downmodulating CXCR4 [28] and efficiently antagonizes SERINC5 to enhance virion infectivity [10,29] which makes it a unique and useful tool for studying the relevance of these functions. In essentially all primate lentiviral Nef proteins, an ExxxLL-based AP interaction domain is critical for down-modulation of CD4, CD28 and CXCR4, as well as for counteraction of SERINC5 [10,30,31]. SIVcol Nef contains a YxxxLL motif at this location and neither reconstitution of ExxxLL nor alteration of LL to AA affected its anti-SERINC5 function [10]. Here, we identified a single tyrosine residue (Y86) as a critical determinant of the ability of SIVcol Nef to antagonize SERINC5 and *Colobus* tetherin. We further show that SIVcol Nef counteracts SERINC5 by a unique mechanism that involves efficient proteasomal degradation of this restriction factor. Despite potent anti-SERINC5 activity, SIVcol Nef hardly promoted HIV-1 replication and cell-to-cell spread in preactivated CD4+ T cells. In comparison, SIVcol Nef significantly enhanced viral replication and CD4+ T cell depletion in *ex vivo* infected human lymphoid tissues and in PBMC cultures that were first infected and stimulated with PHA six days later. However, only the latter effect was dependent on Nef’s activity against SERINC5. Our finding that the highly divergent SIVcol Nef that lacks several established Nef activities counteracts SERINC5 by a distinct mechanism supports an important role of this antiviral factor *in vivo*. However, the effect of Nef to promote virus replication in activated human CD4+ T cells was largely independent of SERINC5 counteraction suggesting that this Nef function might mainly be relevant at limited levels of T cell activation.

## Results

### SIVcol Nef proteins show distinct functional properties

**Fig 1:**
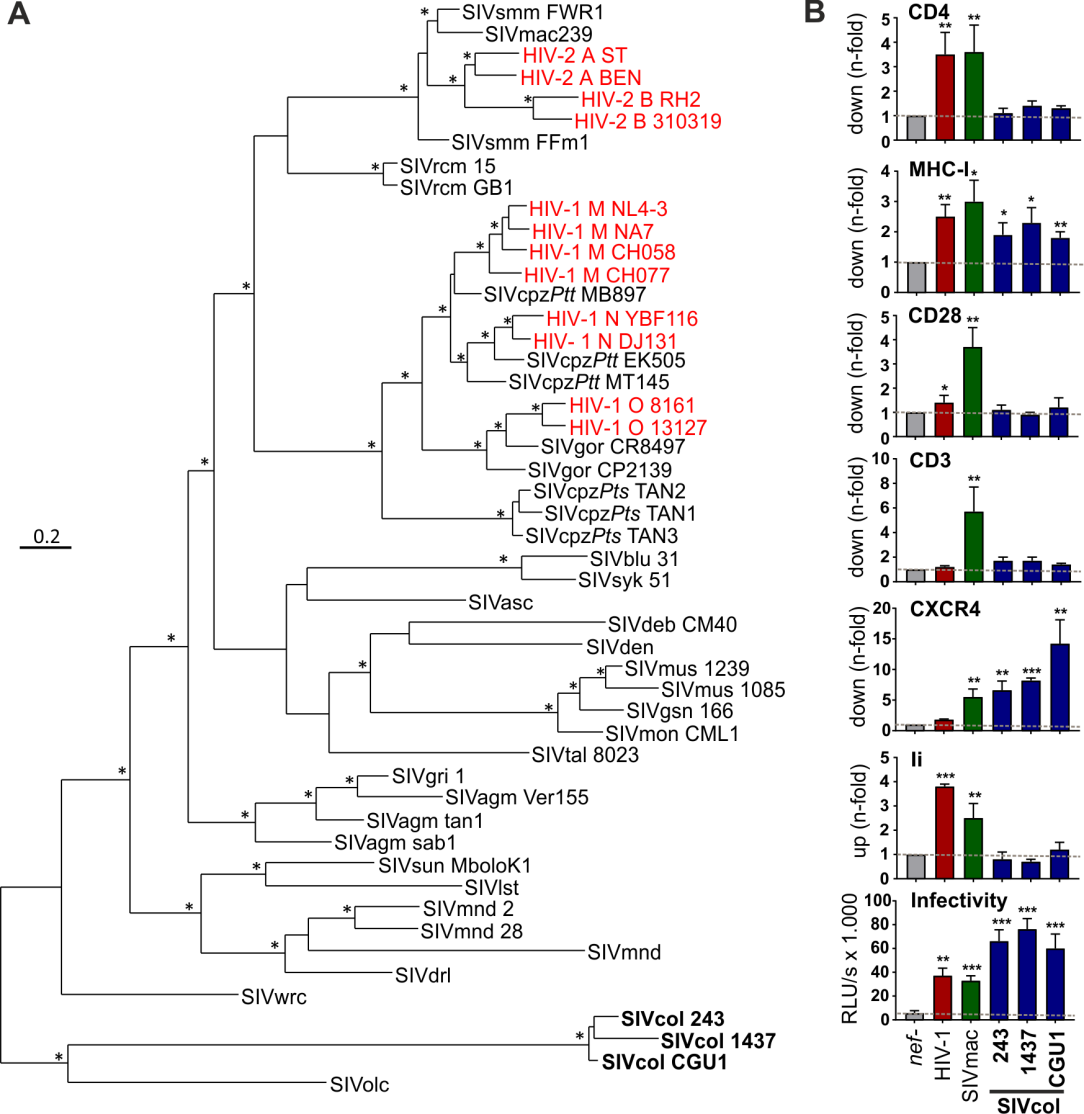
Phylogenetic position and functional activity of SIVcol Nef proteins. (A) Phylogenetic relationship of Nef amino acid sequences from different primate lentiviruses. Stars on branches indicate ≥90 percentage estimated posterior probabilities. The length of a branch indicates the phylogenetic distance to its origin. (B) Modulation of various receptors and viral infectivity enhancement by SIVcol Nefs. To measure receptor modulation, human PBMCs, Jurkat (for CXCR4) or THP-1 cells (for Ii) were transduced with HIV-1 NL4-3 IRES-eGFP constructs containing the indicated *nef* alleles. The mean channel numbers of red fluorescence obtained for cells transfected with the *nef*-defective HIV-1 construct were divided by the corresponding numbers obtained for cells infected with viral constructs coexpressing Nef and eGFP to calculate n-fold down- or up-modulation, respectively. The lowest panel shows the β-galactosidase activity (RLU/s) obtained after infection of P4-CCR5 cells with virus stocks containing 5 ng p24 antigen produced by transfection of HEK293T cells. Results show mean values (+SEM) derived from six to eight measurements in at least two independent experiments. *, p < 0.05; **, p < 0.01; ***, p < 0.001.

Previous analyses of full-length viral genomes showed that SIVcol represents the evolutionarily most isolated primate lentivirus known to date [27]. In agreement with long-term independent evolution, Nef amino acid sequences from SIVcol form a distinct clade that is only distantly related to all other primate lentiviral Nefs including those of SIVolc and SIVwrc infecting olive and western red colobus monkeys (*Procolobus verus* and *Piliocolobus badius*, respectively) (Fig 1A). Expanding the results of previous studies [20,28], we found that SIVcol Nefs show no significant activity in down-modulating CD4, CD28 and TCR-CD3 from the surface of virally infected human CD4+ T cells (Fig 1B). This is not just due to species-specific differences in the Nef target sequences since the cytoplasmic domains of human and colobus monkey CD4 are identical and SIVcol Nef also fails to down-modulate surface proteins carrying the CD3ζ chain derived from colobus monkeys [20]. SIVcol Nefs are also inactive in up-modulating the invariant chain (Ii) associated with immature MHC-II molecules (Fig 1B). This Nef function is usually conserved among different primate lentiviruses [32] and might allow them to suppress effective CD4+ helper T cell responses [33]. In comparison, SIVcol Nefs showed some, albeit modest, activity in decreasing human MHC-I cell surface expression. However, SIVcol Nefs efficiently down-modulated CXCR4 from the surface of Jurkat T cells and strongly enhanced the infectivity of viral particles produced in transfected HEK293T cells (Fig 1B). Importantly, these unusual functional properties were shared by SIVcol Nef proteins derived from three different animals (CM243, CM1437 and CGU1) [20,34] and are thus representative for the highly divergent SIVcol lineage.

### Y86 is critical for efficient SERINC5 antagonism by SIVcol Nef

**Fig 2:**
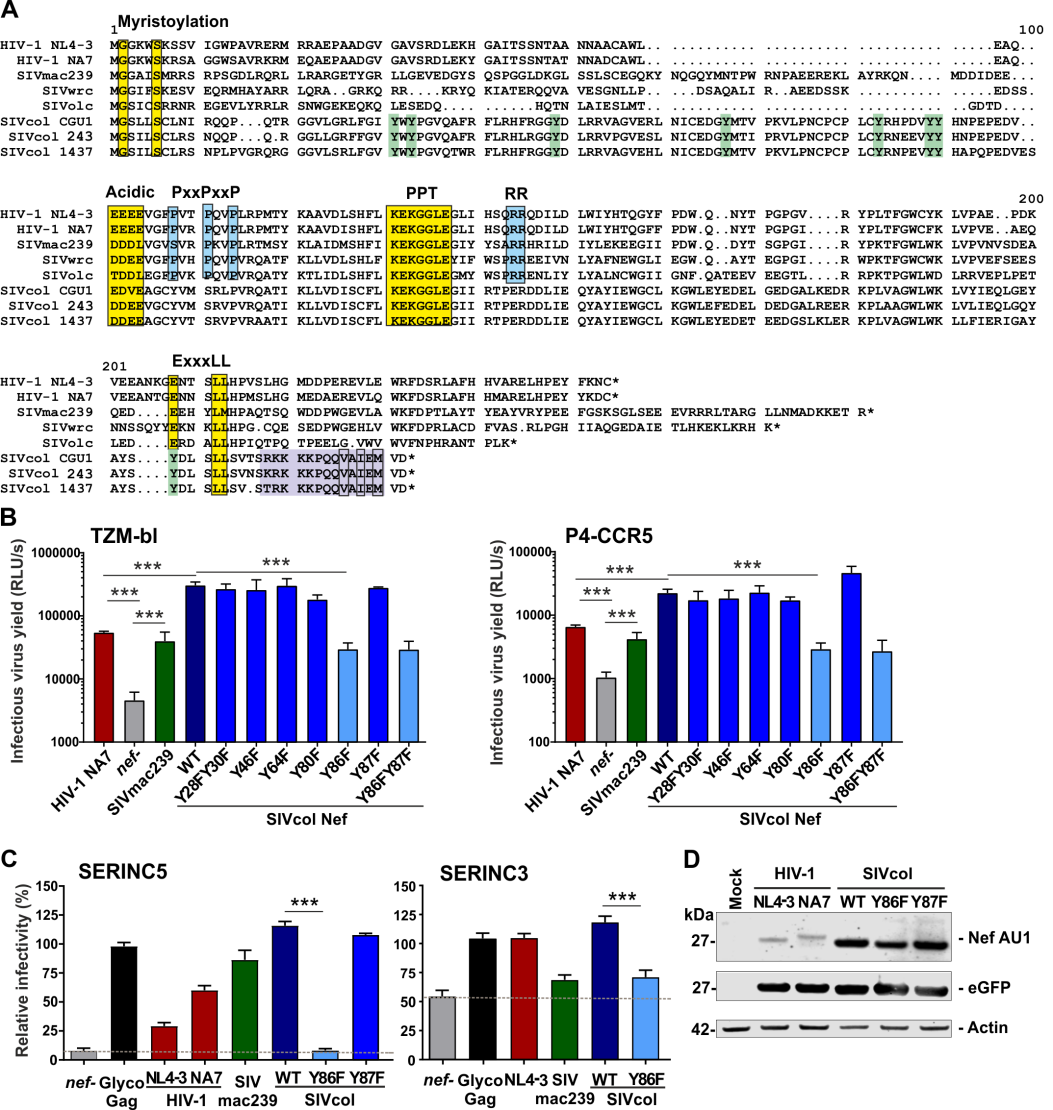
Y86 is critical for SERINC3/5 antagonism by SIVcol Nef. (A) Alignment of the amino acid sequences of HIV and SIV Nef proteins. Dots indicate gaps. Functional amino acid motifs are highlighted. Y residues in SIVcol Nef that were mutated for functional analyses are indicated in green. (B) TZM-bl (left) or P4-CCR5 (right) indicator cells were infected with HIV-1 NL4-3 constructs containing the indicated HIV and SIV *nef* genes or a defective *nef* allele. Infections were performed in triplicate with three different virus stocks. Shown are mean values of the nine measurements +SEM. (C) HEK293T cells were co-transfected with HIV-1 NL4-3 constructs encoding indicated Nef and SERINC5 (left) or SERINC3 (right) or control plasmid. Shown are the mean levels of infectious virus production by the respective IMCs in the presence of transient SERINC5 or SERINC3 expression (+SEM; n = 9) relative to those obtained in cells transfected with the control vector (100%). Results were derived from three experiments, each using triplicate infection of TZM-bl cells to determine infectious virus yield. P-values represent reduction of infectious virus yield by SERINC expression or differences in susceptibility between wt and *nef*-defective HIV-1 IMCs. *, p < 0.05; **, p < 0.01; ***, p < 0.001. (D) Expression of HIV-1 and SIVcol Nef proteins. Western blot analysis of cell lysates following transfection of HEK293T cells with pCG vectors expressing AU1-tagged versions of the indicated Nef proteins. Actin and eGFP are shown as loading and transfection controls, respectively.

In contrast to other primate lentiviral Nef proteins, SIVcol Nef does not require an ExxxLL motif in its C-terminal flexible loop for effective counteraction of SERINC5 [10]. Alignment of Nef amino acid sequences revealed that the ExxxLL adaptor protein (AP) binding site is generally changed to YxxxLL in SVcol but preserved in essentially all other HIV and SIV Nef proteins (Fig 2A). In addition, an SH3-binding motif (PxxP) and the di-arginine (RR) residues in the central core are absent or altered in SIVcol Nefs but conserved in other primate lentiviruses (Fig 2A). Furthermore, SIVcol Nefs contain a truncated C-terminus that shows no sequence similarity to other Nef proteins and contains a stretch of basic amino acids in combination with some hydrophobic residues (VxIxM). Notably, this sequence motif in SIVcol Nef constitutes a predicted linear MAP kinase docking domain [35] that might compensate for the lack of the proline-rich domain, which has also been reported to interact with MAP kinases [36,37]. The above mentioned unusual Nef amino acid sequence features are also found in two additional SIVcol strains from Kibale National park in Uganda [38]. In comparison, the N-terminal myristoylation motif (MGxxxS), an acidic cluster (EEEE) and a stretch of seven amino acids (KEKGGLE) corresponding to the polypurine tract (PPT, on RNA genome level) are conserved in all primate lentiviral Nef proteins including those of SIVcol.

To identify residues critical for anti-SERINC5 activity, we generated a set of HIV-1 NL4-3-based constructs containing the parental or mutant SIVcol CGU1 *nef* alleles harboring alterations in various tyrosine (Y) residues (Fig 2A). We focused on these Y residues since some of them resemble potential tyrosine-based sorting motifs (YxxΦ) that might compensate for the lack of the dileucine-based motif in the C-terminal loop of Nef. Infection of TZM-bl and P4-CCR5 reporter cells with these viral constructs showed that mutation of Y86F alone or in combination with Y87F strongly impaired the ability of SIVcol Nef to enhance virion infectivity, whereas alterations in Y residues at positions 28, 30, 46, 64, 80 and 87 had no significant effect (Fig 2B). Thus, tyrosine 86 plays a key role in the ability of SIVcol Nef to enhance virion infectivity.

To test whether Nef-dependent differences in infection of indicator cell lines (Fig 2B) were due to differential efficacies in SERINC5 antagonism, we cotransfected HEK293T cells with proviral HIV-1 NL4-3 constructs containing various *nef* alleles, as well as SERINC5 expression or control plasmid and measured infectious virus production two days later. As expected from published data [8,9], SERINC5 coexpression reduced infectivity of the *nef*-defective control HIV-1 construct by ∼20-fold and MLV glycoGag potently counteracted this inhibitory effect (Fig 2C). The Y86F mutation disrupted the high anti-SERINC5 activity of SIVcol CGU1 Nef, while Y87F had no significant effect. In agreement with previous reports [8,9], human SERINC3 had only modest inhibitory effects that were counteracted by wt NL4-3 and SIVcol Nef proteins but not by SIVcol Y86F or SIVmac239 Nef (Fig 2C). Western blot analyses showed that the Y86F mutation disrupts the ability of SIVcol Nef to counteract SERINC5 without altering Nef expression levels (Fig 2D).

### SIVcol Nef potently counteracts fish and frog SERINC5

**Fig 3:**
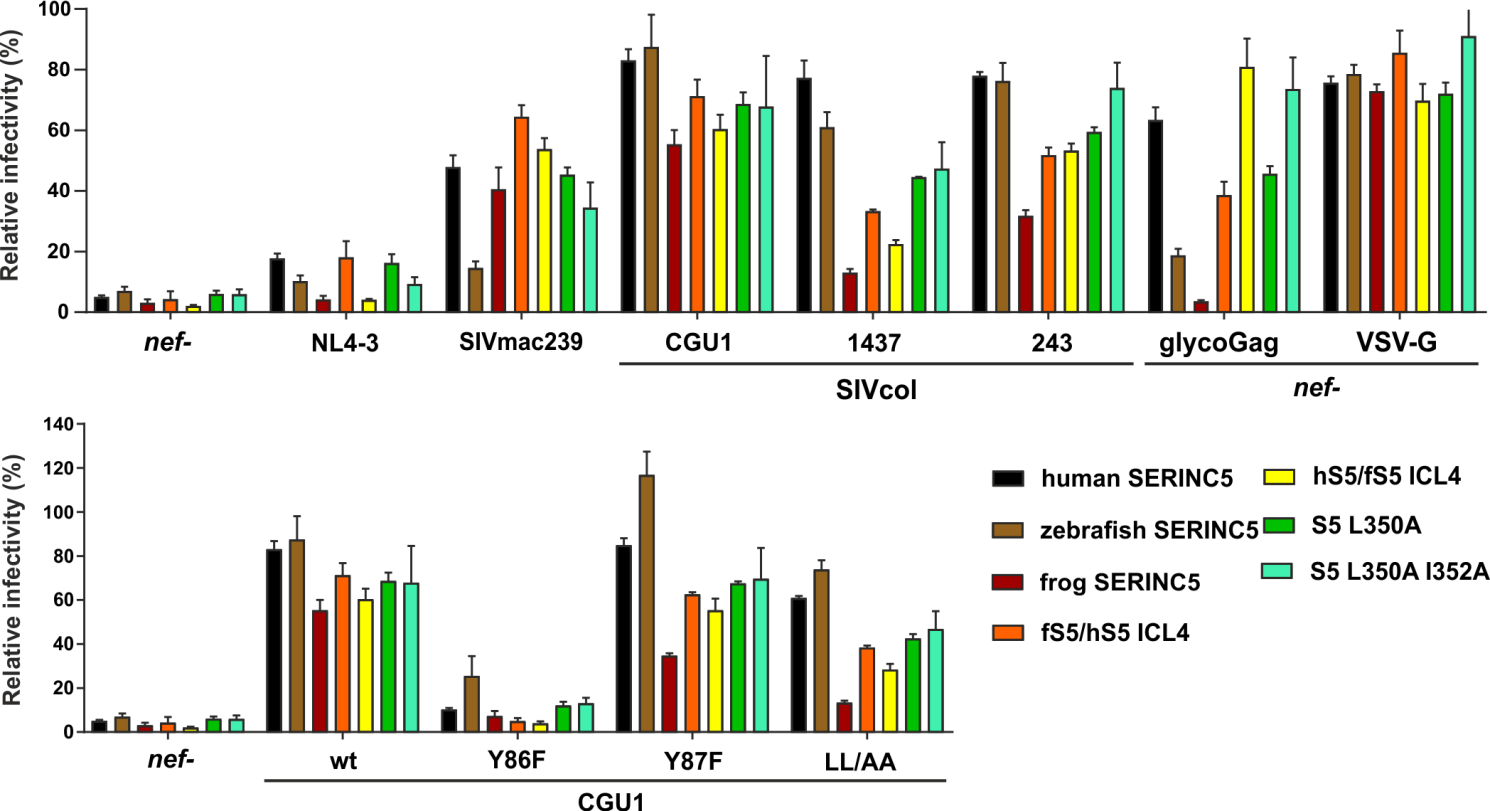
Effect of SIVcol Nef on antiviral activity of vertebrate SERINC5 proteins. Infectious virus yield from HEK293T cells co-transfected with proviral HIV-1 NL4-3 IRES eGFP constructs encoding the indicated *nef* alleles and pBJ5 SERINC expression or control plasmid. Shown are the mean levels of infectious virus production by the respective IMCs in the presence of transient SERINC5 expression (+SEM; n = 9) relative to those obtained in cells transfected with the control vector (100%). The results were derived from three independent experiments, each using triplicate infection of TZM-bl cells to determine infectious virus yield.

In contrast to other antiviral restriction factors, SERINC5 is highly conserved and orthologs from various vertebrate species display antiretroviral activity [10]. Recently, however, it has been shown that frog SERINC5 is resistant to HIV-1 Nef and further demonstrated that the sensitivity is governed by the intracellular loop 4 (ICL4) of the restriction factor [39]. We confirmed these previous findings for HIV-1 NL4-3 Nef, as well as the finding that SIVmac239 Nef is broadly active and capable of counteracting frog SERINC5 (Fig 3). Strikingly, SIVcol CGU1 Nef potently contacted all SERINC5 proteins investigated including the orthologs from both zebrafish and *Xenopus* frog. In agreement with this broad activity, exchanges of ICL4 and substitutions of L350 and I352 to alanines did not affect SERINC5 sensitivity to CGU1 Nef antagonism. In comparison, SIVcol 1437 and 243 Nefs were active against zebrafish but only to a lesser extent against frog SERINC5, while SIVmac239 Nef showed the opposite phenotype (Fig 3). Regions both in and outside of ICL4 seem to contribute to the diminished activity of 1437 and 243 Nef against frog SERINC5. Substitution of Y86F disrupted the effect of SIVcol CGU1 Nef on all SERINC5 variants analyzed, while substitution of Y87F had negligible effects. Substitution of LL/AA slightly attenuated the anti-SERINC5 activity of SIVcol CGU1 Nef, especially against the frog variant (Fig 3, lower). Thus, our results show that the SIVcol Nef is not only a highly potent but also a very broad antagonist of vertebrate SERINC5 proteins.

### Y86 is critical for species-specific tetherin antagonism by SIVcol Nef

**Fig 4:**
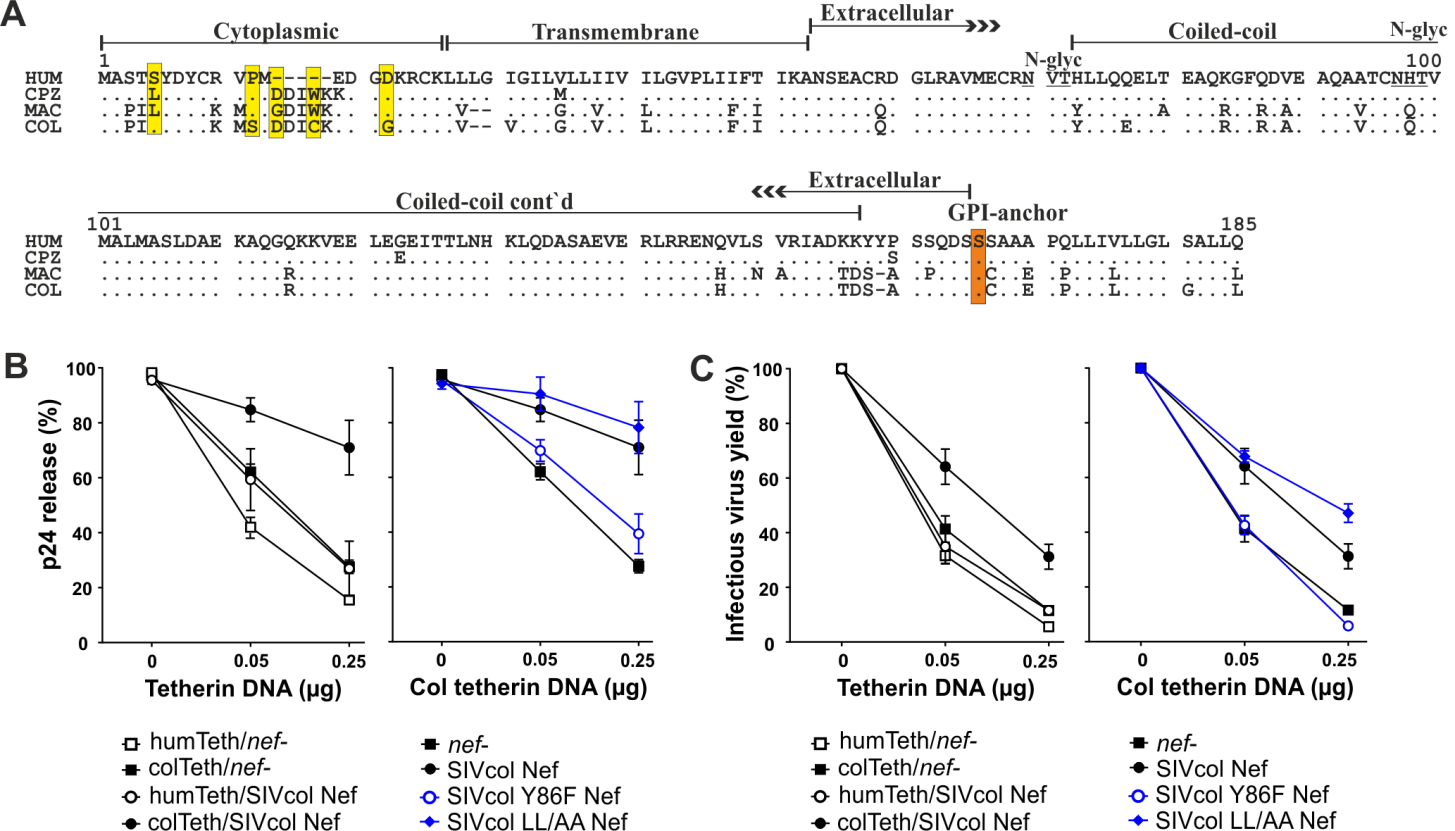
Anti-tetherin activity of SIVcol Nef. (A) Alignment of tetherin amino acid sequences from humans (HUM), chimpanzees (CPZ), rhesus macaques (MAC) and colobus monkeys (COL). Amino acid identity is indicated by dots and gaps by dashes. Differences between COL and other tetherins in the TM domain are highlighted by yellow boxes. Some known domains, the serine GPI anchor site (red box), and two potential glycosylation sites (underlined) are indicated. (B, C) Release of p24 capsid antigen (B) and infectious virus yield (C) from HEK293T cells following transfection with a Δ*vpu*Δ*nef* proviral HIV-1 NL4-3 construct, SIVcol Nef or GFP only (nef*) expression constructs, and varying amounts of plasmids expressing human or *Colobus* tetherin. Virus release was determined by ELISA of cell-free and cell-associated p24 antigen and infectious virus was determined by infection of TZM-bl indicator cells. Both are shown as percentages of those detected in the absence of tetherin (100%) and all values were derived from triplicate experiments ± SEM.

Many primate lentiviruses use Nef to counteract the restriction factor tetherin [21–24]. This effect is frequently species-specific and the cytoplasmic domain of *Colobus guereza* tetherin targeted by Nef differs at several positions from those of other primate tetherin orthologs (Fig 4A). To determine the anti-tetherin activity of SIVcol, we measured p24 release and infectious virus yield from HEK293T cells following cotransfection of a *vpu*-deleted (Δ*vpu*) HIV-1 proviral construct with different *nef* alleles and tetherin expression plasmids. Our data showed that SIVcol Nef increases p24 release (Fig 4B, left) and infectious virus yield (Fig 4C, left) in the presence of *Colobus* (COL) tetherin. As anticipated, SIVcol Nef did not efficiently counteract the human tetherin ortholog (Fig 4B and 4C) that differs from COL tetherin by six residues and a five amino acid deletion in the cytoplasmic part (Fig 4A). The activity of SIVcol Nef against *Colobus* tetherin was disrupted by the Y86F substitution (Fig 4B and 4C). It has been shown, that the ExxxLL motif is critical for recruitment of AP-2 adaptor protein complexes and counteraction of tetherin by SIVmac Nef [24]. In contrast, mutation of the two leucines (YxxxLL to YxxxAA) did not impair the anti-tetherin activity of SIVcol Nef (Fig 4B and 4C).

### SIVcol Nef suppresses T cell migration and actin remodeling

**Fig 5:**
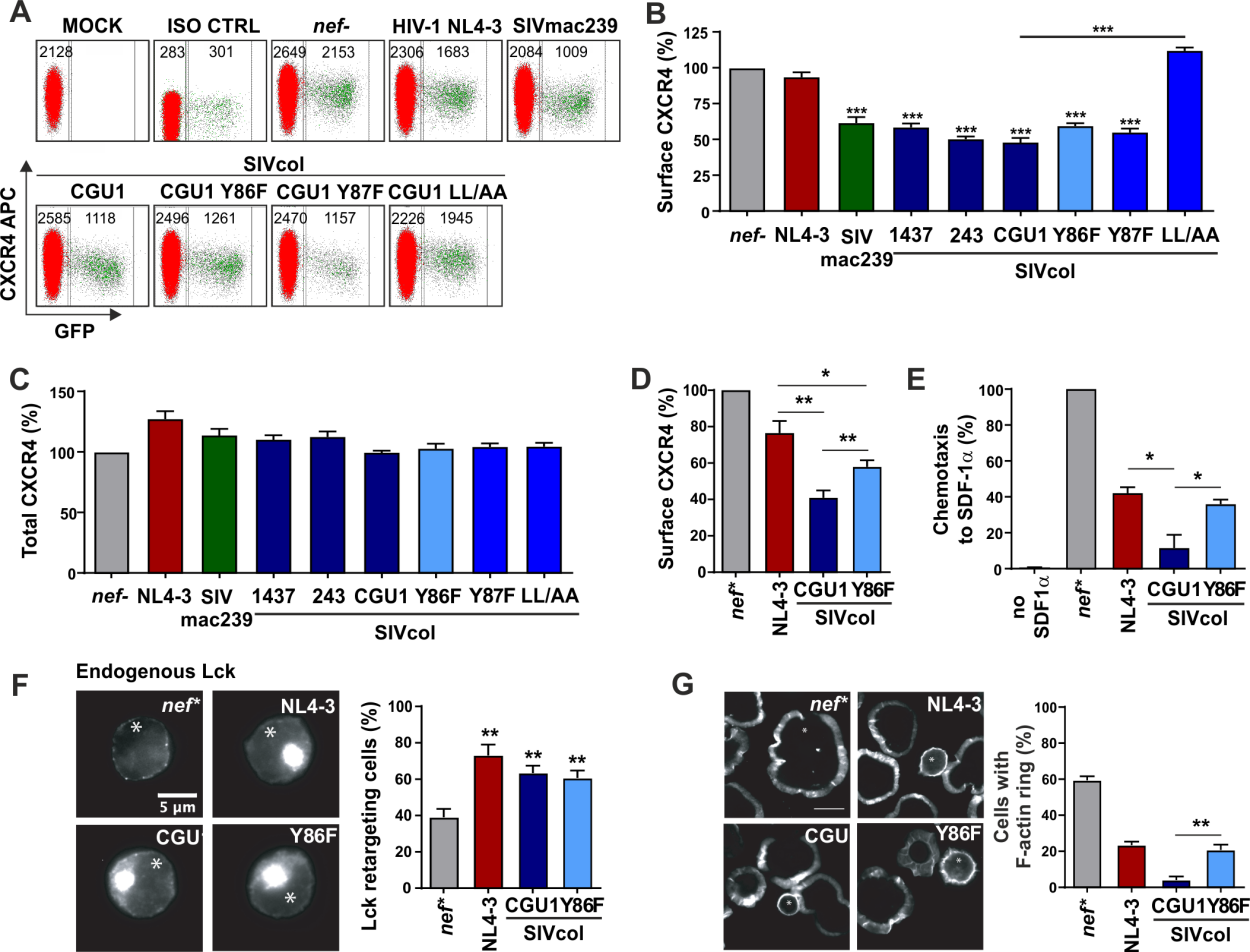
Modulation of T cell properties by SIVcol Nef variants. (A) Human PBMCs were transduced with VSV-G pseudotyped HIV-1 NL4-3 constructs coexpressing the indicated *nef* alleles and eGFP and assayed for surface expression of CXCR4 two days later. The mean APC fluorescence intensity values used to calculate receptor modulation are indicated. (B, C) Quantitative assessment of surface (B) and total (C) cellular levels of CXCR4 in infected PBMCs. Values give means + SEM derived from four donors. (D) Cell surface CXCR4 expression and chemotaxis (E) of Jurkat T (CCR7) cells. CXCR4 modulation was calculated in transfected GFP expressing cells for each condition to their respective untransfected cells (100%). Chemotaxis was determined in a transwell system (5μm pore size) towards SDF-1α (10 ng/mL) for 2 h after starvation in hunger medium (0.5% FCS). The percentage of migrated cells was calculated from % GFP expressing cells after migration relative to % GFP expressing cells given as input per condition. Shown are mean values with standard error mean of three independent experiments each performed in duplicates. (F) Representative widefield images and frequency of Lck retargeting assay in transient Nef expressing Jurkat-TAg T cells and control. Cells were plated onto coverslips, fixed, permeabilized and stained for endogenous Lck. The frequency of cells showing retargeting of Lck to the perinuclear region was determined by counting at least 100 cells per condition. (G) Representative widefield images and quantification of peripheral F-actin ring formation by Jurkat-TAg T cells upon activation on anti-CD3 coated coverslips. The right panel shows quantification of samples shown in the images counting at least 100 cells per condition. (*) Asterisks indicate GFP-positive cells. Bar diagrams show mean values with standard deviation of three independent experiments. Scale bar = 5 μm. *, p < 0.05; **, p < 0.01; ***, p < 0.001.

To further define the determinants of SIVcol Nef function, we transduced human peripheral blood mononuclear cells (PBMCs) with HIV-1 NL4-3 based IRES-eGFP constructs containing different *nef* alleles and performed flow cytometric analysis to determine CXCR4 surface expression (Fig 5A). All three parental SIVcol Nefs efficiently down-modulated CXCR4 in primary human cells (Fig 5B). This effect was disrupted by mutation of YxxxLL to YxxxAA in the C-terminal region of SIVcol CGU1 Nef but hardly affected by substitution of Y86F or Y87F (Fig 5B). All SIV Nef proteins down-modulated CXCR4 from the cell surface without significantly altering the overall levels of CXCR4 expression (Fig 5C). Thus, in contrast to counteraction of SERINC5 and tetherin, down-modulation of the CXCR4 chemokine receptor by SIVcol Nef involves a dileucine motif, a feature that is shared by other primate lentiviruses [14]. It has been shown that Nef-mediated down-modulation of CXCR4 inhibits migration of virally infected T cells to the chemokine stromal derived factor 1 (SDF-1) [14]. We confirmed that potent down-modulation of CXCR4 from the surface of Jurkat T cells (Fig 5D) was associated with strongly inhibited cellular migration towards SDF-1α (Fig 5E). Both effects were attenuated but not fully disrupted by the Y86F mutation.

HIV and SIV Nefs induce intracellular accumulation of the Src kinase Lck [40]. To determine whether SIVcol Nef also exerts this activity, we stained Jurkat-TAg T cells transiently transfected with Nef expression constructs for endogenous Lck (Fig 5F, left). Analyses of ≥100 cells per construct showed that SIVcol Nef promotes retargeting of Lck to the perinuclear region almost as efficiently as HIV-1 Nef, and this activity was not affected by the Y86F change (Fig 5F, right). Finally, we analyzed whether SIVcol Nef suppresses the polymerization of actin into a circumferential ring (F-actin ring) upon TCR-CD3 triggering, as reported for HIV-1 and SIV Nef proteins [11,41]. Although SIVcol Nefs lack the CD3 down-modulation function [20] they inhibited F-actin ring formation with high efficiency (Fig 5G). The Y86F change attenuated this activity but mutant SIVcol Nef still remained as active as HIV-1 NL4-3 Nef. Thus, although SIVcol Nefs lack a variety of T cell modulatory functions exerted by other Nef proteins, such as down-modulation of CD3, CD4 and CD28, they are capable of interfering with T cell migration, actin polymerization and Lck distribution.

### SIVcol Nef induces proteasomal degradation of SERINC5

**Fig 6:**
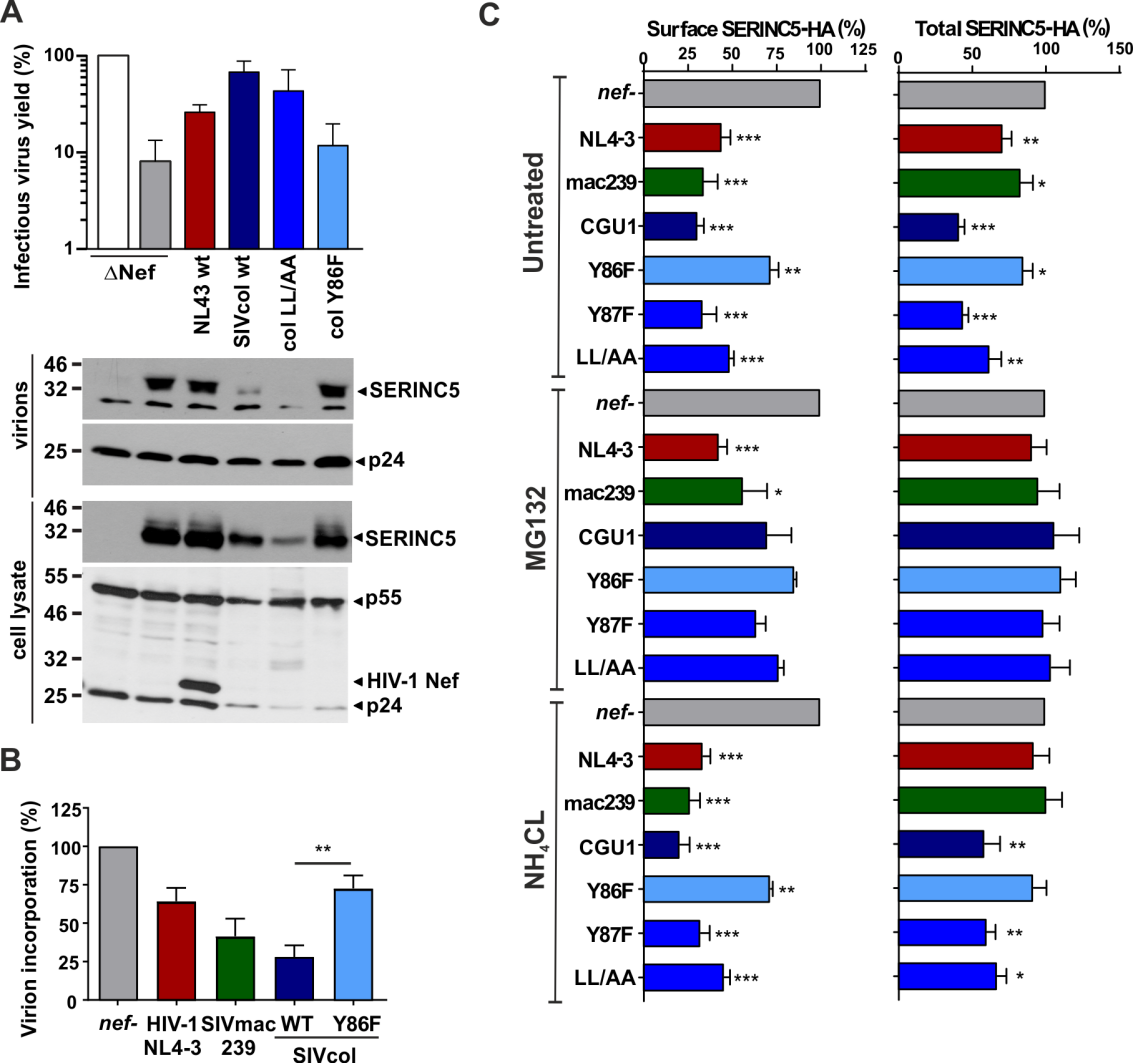
Effect of SIVcol Nef on SERINC5 expression and virion incorporation. (A) The upper panel shows the infectivity of HIV-1 particles produced in HEK293T cells relative to the *nef*-defective control vector (100%). The lower panel shows Western blot analysis of lysates of the corresponding producer cells and purified virions. (B) Quantification of SERINC5 levels in virions determined by densitometry of Western blot analysis. Depicted are means (+SEM) from at least four independent experiments. (C) Surface and total cellular SERINC5 levels in Jurkat T cells containing an HA-coding sequence in exon 8 of the *serinc5* alleles (corresponding to the predicted fourth extracellular loop of SERINC5) introduced by CRISPR/Cas9-assisted gene editing infected with NL4-3 IRES eGFP expressing the indicated Nef proteins. Cells were left untreated or treated with MG132 or ammonium chloride, which inhibit proteasomal and lysosomal degradation, respectively. Protein levels were determined at 2 days post-infection. Shown are means of four experiments (+SEM). *, p < 0.05; **, p < 0.01; ***, p < 0.001.

SIVcol CGU1 Nef is more active in counteracting SERINC5 than other primate lentiviral Nef proteins and seems to use a distinct mechanism that involves Y86 but not the dileucine motif in the C-proximal region [10]. To further characterize the underlying mechanism(s), we examined the effect of SIVcol Nef on SERINC5 expression and virion incorporation. Western blot analyses showed that wt SIVcol Nef not only reduced virion incorporation but strongly diminished the cellular protein levels of SERINC5 (Fig 6A). Mutation of Y86F but not of YxxxLL to YxxxAA (LL/AA) impaired this effect. In contrast to SIVcol Nef, the HIV-1 NL4-3 Nef enhanced virion infectivity without affecting SERINC5 expression levels (Fig 6A). Quantitative analyses revealed that wt SIVcol Nef reduced virion incorporation of SERINC5 by 75%, whereas NL4-3 and SIVmac239 Nefs only achieved 40% and 60% reduction, respectively (Fig 6B). The Y86F mutation significantly abrogated the ability of SIVcol Nef to prevent degradation, virion incorporation and down-modulation of SERINC5 (Fig 6A–6C). Addition of MG132, an inhibitor of cellular proteasome activity but not the lysosomotropic agent NH_4_Cl diminished SIVcol Nef dependent reduction of SERINC5 expression levels (Fig 6C) suggesting that this Nef enhances virion infectivity at least in part by inducing proteasomal degradation of SERINC5.

**Fig 7:**
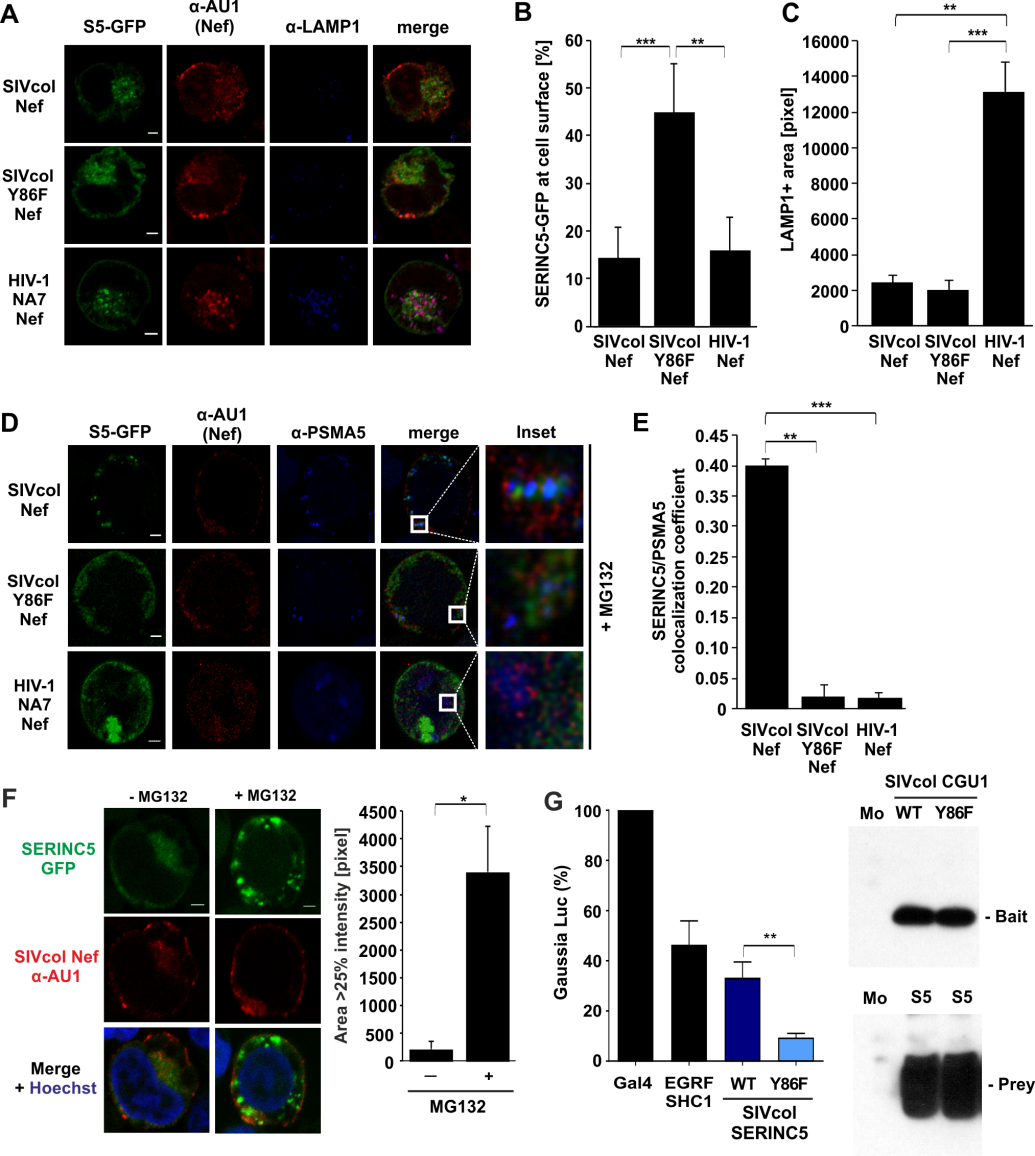
SIVcol Nef relocalizes SERINC5 to the proteasome by an Y86 dependent mechanism. (A) Representative Laser scanning confocal microscopy images of JTAg SERINC3/5 knock-out cells transfected with SERINC5–GFP (S5-GFP; green) alone or together with the indicated AU1-tagged Nef proteins (red). Endogenous lysosomes were stained with an anti-LAMP1 antibody (blue). (B) Quantification of internal SERINC-GFP fluorescence versus surface SERINC-GFP fluorescence from (A) displayed as means of n = 5 (+SD). (C) Quantification of the pixel area of LAMP1 staining from (A) in triplicates (+SD). (D) Representative Laser scanning confocal microscopy images of JTAg SERINC3/5 knock-out cells transfected with SERINC5–GFP (green) together with indicated AU1-tagged Nef (red) and endogenous proteasomes (anti-PSMA5, blue). Insets show magnifications of the highlighted areas. Size bar, 2 μm. (E) Calculation of Pearson’s co-localization coefficients using Costes thresholds for PSMA5 (proteasome) and SERINC5-GFP for images in (D). Displayed as means of triplicates (+SD). (F) Representative Laser scanning confocal microscopy images (left) and quantitative analysis (right) of JTAg SERINC3/5 knock-out cells transfected with SERINC5–GFP (green) together with AU1-tagged SIVcol Nef wt (red) and either treated with MG132 (10 μM for 3 h) or mock treated. Nuclei are stained with Hoechst (blue). (G) HEK293T B0166 MaMTH reporter cells were co-transfected with 25 ng Bait (Nef) and 25 ng Prey (SERINC5) DNA. After 24 h, protein expression was induced by adding 0.5 μg/ml tetracycline and 40 h post-transfection, *Gaussia* luciferase luminescence (left) was measured in three independent experiments, each using triplicates of transfection (+SEM) to determine the level of protein interaction as compared to positive control (transcription factor only, Gal4) and EGFR/SHC1 proteins known to interact with each other. Negative control cells co-transfected with each Nef Bait with Pex7 Prey were used to substract non-specific background interaction signal. Western blot (right) shows the expression of V5-tagged SIVcol Nef Baits (WT or Y86F) and FLAG-tagged SERINC5 Prey (S5) constructs in HEK293T B0166 cells. Mo = mock cells. *, p < 0.05; **, p < 0.01; ***, p < 0.001.

Confocal microscopy analyses confirmed that HIV-1 and SIVcol Nef relocalized GFP-tagged SERINC5 from the cell surface to intracellular structures (Fig 7A) and this effect was strongly attenuated by the Y86F mutation (Fig 7B; supplemental S1–S4 Movies). Although HIV-1 and SIVcol Nef proteins both removed SERINC5 from the plasma membrane, only HIV-1 Nef induced accumulation of LAMP-1 positive vesicles and showed significant colocalization with this lysosomal marker (Fig 7A and 7C). In contrast, wt SIVcol Nef but not the Y86F mutant or HIV-1 NA7 Nef induced significant colocalization of SERINC5 with PSMA5, a component of the 20S core proteasome complex (Fig 7D and 7E). Recruitment of SERINC5 to the proteasome was examined in the presence of MG132 (Fig 7D) since SIVcol Nef induced a drastic reduction of the SERINC5 signal in the absence of this inhibitor of proteasomal degradation (Fig 7F). It has been previously reported that SERINC5 downmodulation from the cell surface by HIV-1 Nef involves the autophagy/lysosomal pathway [42]. Our results clearly demonstrate that SIVcol Nef counteracts SERINC5 by a distinct mechanism involving proteasomal degradation.

To clarify whether the Y86F mutation affects binding of SIVcol Nef to SERINC5, we used the mammalian-membrane two-hybrid assay (MaMTH) allowing to detect even transient interactions between membrane proteins in living human cells [43]. SIVcol Nef and SERINC5 reporter fusions were efficiently expressed (Fig 7G). Coexpression of wt SIVcol Nef and SERINC5 resulted in marked *Gaussia* luciferase reporter activity compared to controls indicating interaction of both proteins. Signal intensity was significantly reduced by the Y86F mutation (Fig 7G) suggesting that this residue is required for efficient interaction between SIVcol Nef and SERINC5 at cellular membranes.

### SERINC5 antagonism does not enhance viral replication in preactivated CD4+ T cells

**Fig 8:**
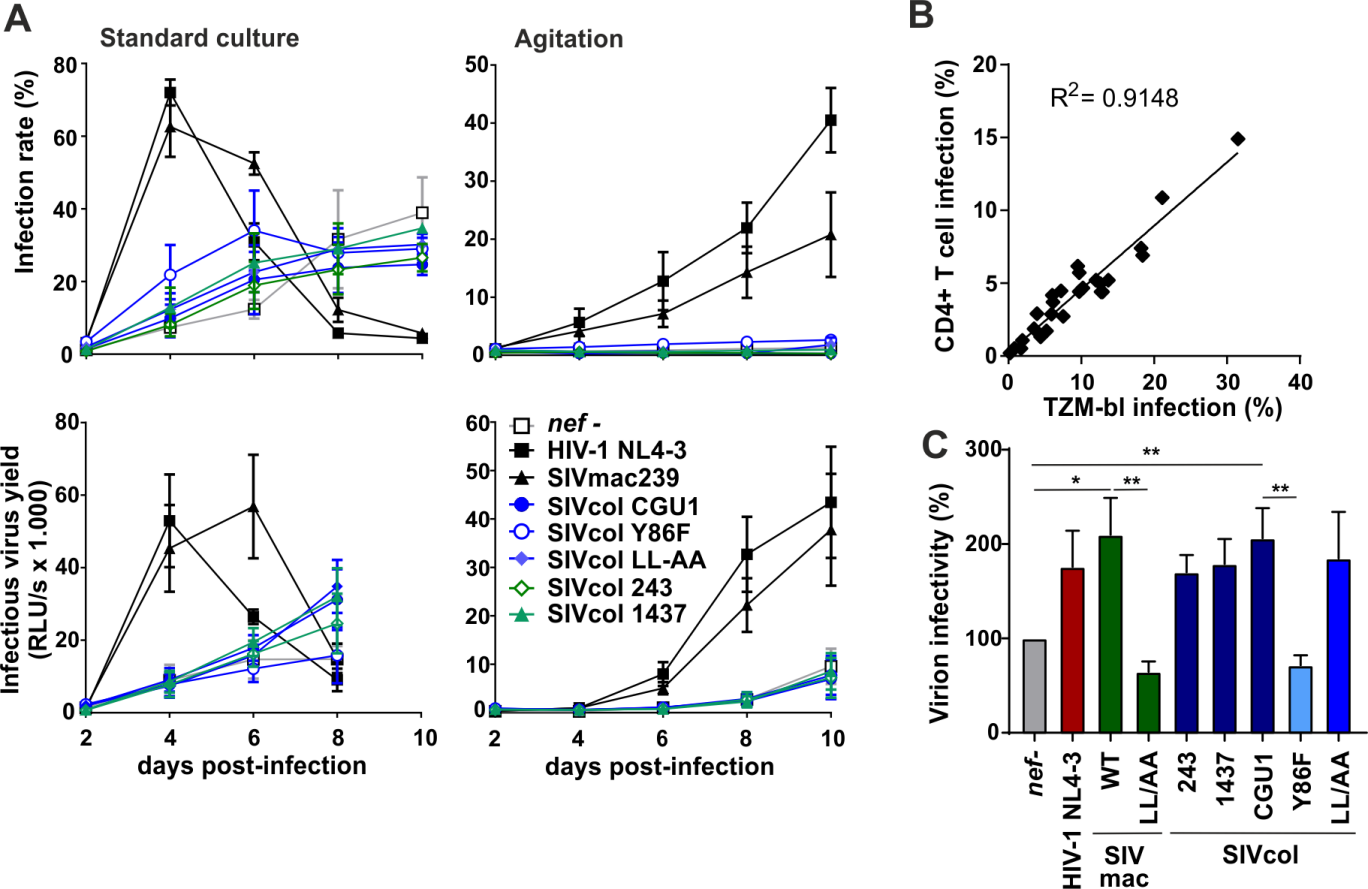
SERINC5 antagonism does not enhance viral replication in primary human CD4+ T cell cultures. (A) Stimulated primary CD4+ T cells were transduced with equal p24 amounts of VSV-G pseudotyped NL4-3 viruses carrying the indicated HIV/SIV *nef* gene or mutant thereof. Cells were maintained in culture for up to 10 days post-transduction under static conditions (left panel) or with agitation (right panel) to limit cell-to-cell viral spread. Every 2 days, samples of cell culture medium from triplicate wells were harvested to determine infectious virus yield by TZM-bl reporter cell assay and CD4+ T cell infection rates were assessed by intracellular p24 staining followed by flow cytometric analysis. Data shown represents measurements obtained from 3 donors (mean ±SEM). (B) Correlation between the infection rates of TZM-bl and primary CD4+ T cells infected with HIV-1 IRES-eGFP *nef* recombinants produced in the presence of transiently expressed SERINC5 in HEK293T cells. (C) The infectious virus and p24 antigen yields at 4, 6 and 8 days post-infection in the standard CD4+ T cell infection experiments shown in panel A were determined by TZM-bl infection and p24 antigen ELISA, respectively. Virion infectivity normalized for p24 content is shown relative to the *nef*-defective HIV-1 construct (100%). Shown are average values obtained at three time points for the three blood donors (+SEM). *p < 0.05; **p < 0.01.

To assess whether the ability of Nef to counteract SERINC5 enhances viral replication, we transduced pre-activated CD4+ T cells with HIV-1 NL4-3 expressing wt or Y86F SIVcol or control HIV-1 and SIVmac *nef* alleles. Pseudotyping with the VSV-G envelope protein allowed to achieve similar infection rates during the first round of infection as it bypasses the effect of Nef on virion infectivity [44]. We found that recombinant viruses containing intact HIV-1 NL4-3 or SIVmac239 *nef* genes spread with higher efficiency and accelerated kinetics than the *nef*-defective virus (Fig 8A, upper left). In contrast, SIVcol *nef* genes did not enhance viral replication in human CD4+ T cells. Similarly, HIV-1 and SIVmac strongly enhanced infectious virus production by infected CD4+ T cell cultures, while SIVcol Nefs had only marginal effects (Fig 8A, lower left). Thus, the ability of Nef to enhance virion infectivity by counteraction of SERINC5 was not associated with increased viral replication in pre-activated primary human T cells.

It has been established that cell-to-cell viral transfer through the formation of virological synapses between donor and target cells is the predominant mode of HIV spread and replication [45,46]. To determine whether SERINC5 counteraction might affect viral spread under conditions of reduced cell-to-cell virus transmission, we maintained the infected CD4+ T cell cultures under continuous agitation as previously described [46]. In agreement with published data [45,46], reduced cellular contacts strongly delayed virus spread and infectious virus production compared to experimental conditions allowing effective cell-to-cell viral transmission (Fig 8A, right panels). Expectedly, significant viral spread and infectious virus production were observed in cultures infected with viruses expressing HIV-1 or SIVmac Nef proteins. In contrast, lack of Nef expression or expression of wt or Y86F mutant SIVcol Nef proteins was associated with lack of viral replication if cells were agitated. Thus, cell-free virus replication seems to be even more dependent on functional Nef expression than cell-to-cell virus spread.

To further determine why SERINC5 antagonism failed to significantly enhance HIV-1 replication in these settings, we examined whether the effects of this restriction factor and its antagonism on virion infectivity might be less pronounced in PHA-stimulated human CD4+ T cells than in TZM-bl indicator cells. To assess possible target cell dependencies, we cotransfected HEK239T cells with recombinant HIV-1 NL4-3 proviral constructs containing a broad spectrum of *nef* alleles and a SERINC5 expression vector. Nef proteins that efficiently enhanced virion infectivity for TZM-bl cells by counteracting SERINC5 also potently promoted infection of primary CD4+ T cells (Fig 8B). To assess whether SERINC5 affects the infectiousness of viral particles produced by infected primary CD4+ T cells, we determined infectious virus and p24 yield in the supernatant of the cultures shown in Fig 8A. The presence of *nef* alleles capable of counteracting human SERINC5 was associated with increased infectiousness of HIV-1 particles produced by CD4+ T cells (Fig 8C). However, the enhancing effect of functional HIV-1, SIVmac and SIVcol Nef proteins on HIV-1 particle infectivity was weaker (about 2- fold) compared to the up to 100-fold increase achieved in transient HEK293 T transfection assays and in Jurkat T cells (e.g. Fig 2C) [8–10].

### SERINC5 antagonism promotes HIV-1 infectivity under conditions of limited T cell activation

**Fig 9:**
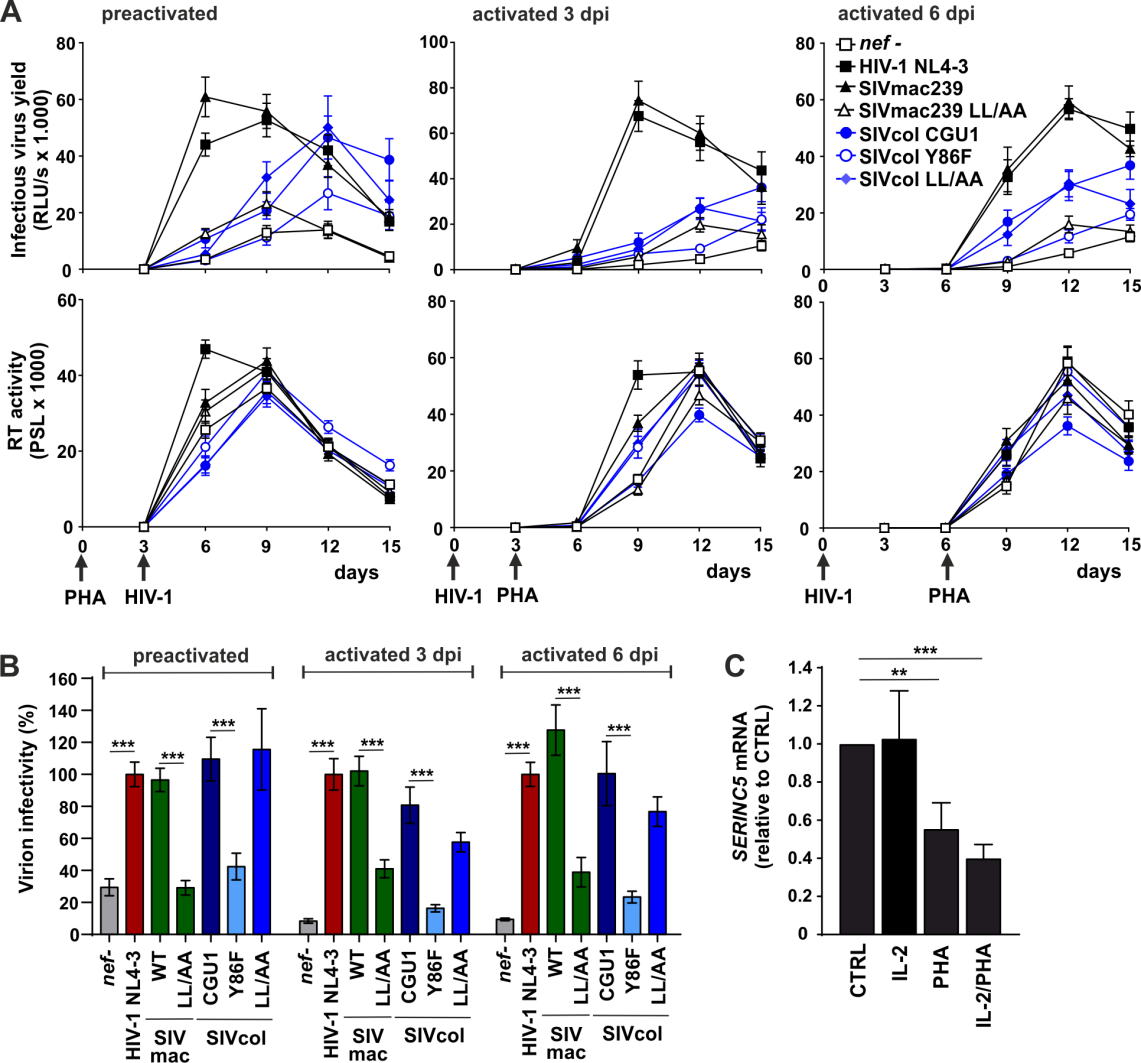
Effect of SERINC5 antagonism on HIV-1 replication in PBMC cultures. (A) Human PBMC were transduced with equal p24 amounts of VSV-G pseudotyped NL4-3 viruses carrying the indicated HIV/SIV *nef* gene or mutant thereof. Cells were activated with IL-2 and PHA either 3 days prior to transduction (preactivated) or 3 days and 6 days post infection (dpi). Every 3 days, samples of cell culture medium from triplicate wells were harvested to determine infectious virus yield and RT activity. (B) Relative infectivity of virions produced in PBMCs. Results were calculated by dividing infectious virus yield values obtained from TZM-bl reporter infectivity assay by corresponding RT activity values measured by RT radioactivity-based assay of the cell culture supernatants from day 12. (C) SERINC5 mRNA levels in PBMCs cultured in the presence of 2 μg/ml PHA and/or 10 ng/ml IL-2. Gene expression was measured through qRT-PCR and the values were normalized to internal GAPDH control as well as unstimulated PBMCs (CTRL) of the corresponding donor. Data shown represents measurements obtained from 4 donors (mean ±SEM). **, p < 0.01; ***, p < 0.001.

It has been shown that Nef is most effective in enhancing viral replication if primary PBMC cultures are first infected and stimulated several days later [47,48]. In addition, it has been reported that resting PBMCs express higher levels of SERINC5 than PHA-activated cells [49]. To determine whether Nef-mediated counteraction of SERINC5 might be more relevant under conditions of limited activation, we isolated PBMCs from five donors and infected them prior to or after PHA activation. The HIV-1 NL4-3 and SIVmac239 Nef proteins efficiently increased infectious HIV-1 yield, while expression of the SIVcol CGU1 and LL/AA Nefs was associated with substantially delayed kinetics in prestimulated PBMCs and cultures that were first infected and stimulated three days later (Fig 9A, upper). Notably, the SIVcol CGU1 and LL/AA Nefs that counteract SERINC5 moderately increased infectious virus yield compared to the Y86F Nef during later time-points. When the PBMC cultures were stimulated 6 days post-infection, the CGU1 and LL/AA SIVcol Nefs resulted in a phenotype intermediate between wt and *nef*-defective HIV-1, while the Y86F Nef had only marginal effects (Fig 9A, upper right). The impact of functional Nef on infectious virus yield was more pronounced than on RT activity in the culture supernatants (Fig 9A, lower). To assess the effect of the various Nef proteins on virion infectivity, we divided the infectious virus yield by the corresponding RT activities. In agreement with the results obtained in transient transfection assays and in purified CD4+ T cells, the NL4-3, SIVmac239, SIVcol CGU1 and LL/AA Nefs Nefs significantly enhanced virion infectivity, while the SIVmac239 LL/AA and SIVcol Y86F Nefs had little if any effect (Fig 9B). Notably, the magnitude of enhancement compared to the *nef*-defective control HIV-1 construct was less pronounced in pre-activated PBMCs (~3-fold) than in cells stimulated after virus infection (7-15-fold). We found that the levels of SERINC5 mRNA expression in PBMCs are significantly reduced by PHA-stimulation (Fig 9C). Altogether, these results suggest that SERINC5 has stronger inhibitory effects on virion infectivity in CD4+ T cells that are not fully activated and might express higher levels of SERINC5. Furthermore, our data show that Nef activities other than antagonism of SERINC5 also contribute to viral spread and replication in primary human cells.

### SIVcol Nef promotes viral spread in lymphoid tissue independently of anti-SERINC5 activity

**Fig 10:**
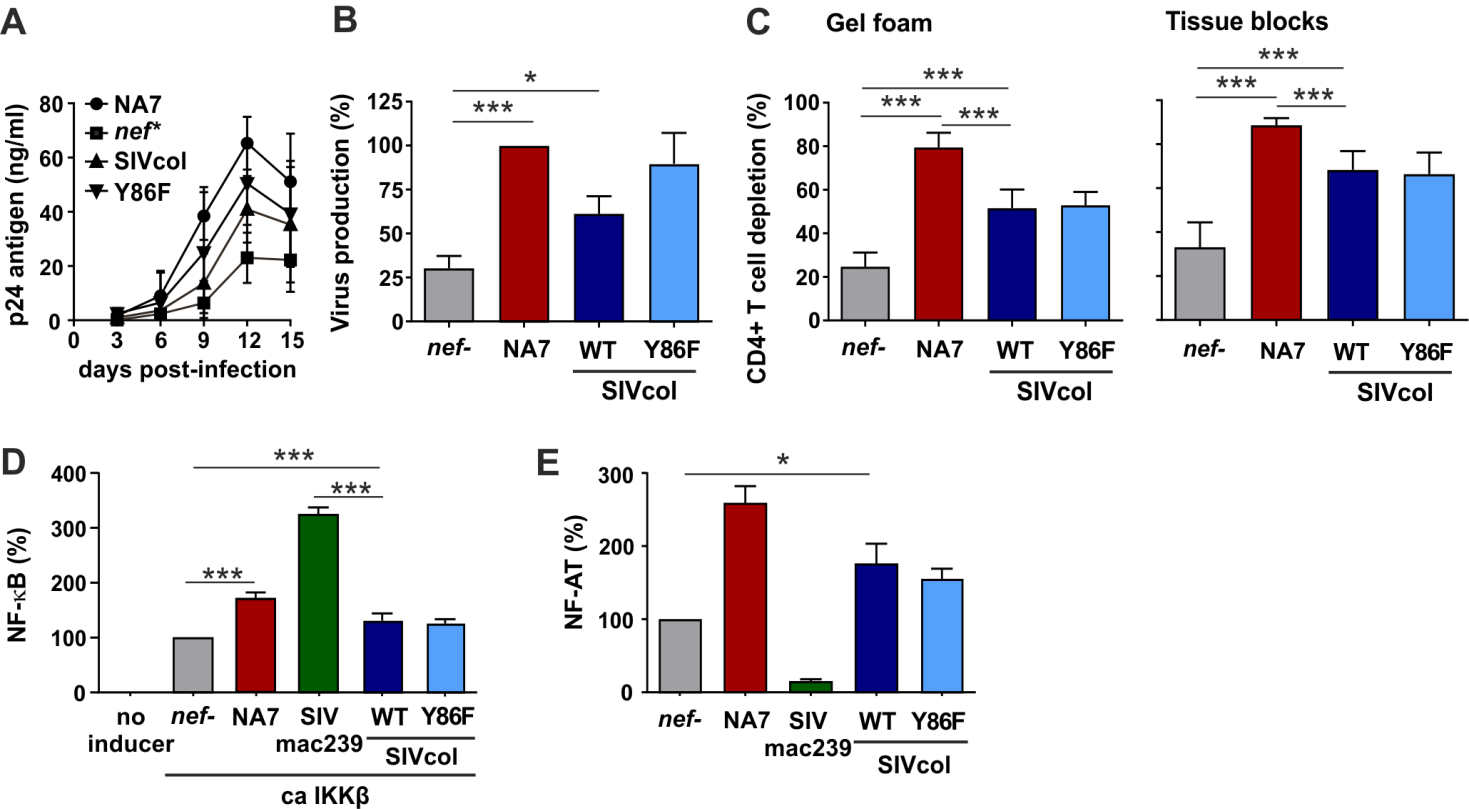
Effect of SIVcol Nef on viral replication in *ex vivo* HLT and NF-κB or NF-AT activity. (A) Replication kinetics of HIV-1 constructs containing the indicated *nef* alleles in blocks of human lymphoid tissues *ex vivo*. Panels A to C show mean values (±SEM) obtained using tissues from seven different donors. (B) Cumulative virus production over 15 days of infection by the indicated HIV-1 constructs (see panel A) relative to the replication of the HIV-1 construct containing the NA7 *nef* allele (100%). (C) Levels of CD4+ T cell depletion in the tissue blocks (left) and cells that migrated in the gel foams (right) at the end of culture at 15 days post-infection. (D) HEK293T cells were cotransfected with a firefly luciferase reporter construct under the control of three NF-κB binding sites, a *Gaussia* luciferase construct for normalization, and expression vectors for a constitutively active mutant of IKKβ and the indicated Nef variants. Luciferase activity was determined 40 h post-transfection. The mean value of 9 transfections + SEM is shown. (E) Jurkat cells stably transfected with an NF-AT-dependent luciferase reporter gene were transduced with the indicated HIV-1 Nef-IRES-eGFP variants. The levels of luciferase activity were determined at 16 h post-stimulation. Shown are average values (+SD) derived from triplicate transductions relative to the *nef*-defective control HIV-1 construct (100%). Similar results were obtained in two independent experiments. *, p < 0.05; ***, p < 0.001.

It has been shown that HIV-1 Nef promotes viral replication and cytopathicity in *ex vivo* infected human lymphoid tissue (HLT) [50]. This experimental system is based on tonsillar explants and maintains the mixed cell populations characteristic of lymphoid tissues, the major site of viral replication in HIV-1-infected individuals. Notably, *ex vivo* HLT supports productive HIV-1 infection in the absence of exogenous stimulation and may thus allow detection of Nef effects on T cell activation that are missed under experimental conditions requiring prior stimulation. In agreement with published data [50], disruption of the *nef* gene significantly reduced HIV-1 replication and CD4+ T cell depletion (Fig 10A–10C). We found that SIVcol Nef resulted in a phenotype intermediate between *nef*-defective and wt HIV-1 infection (Fig 10A–10C). On average, however, the levels of p24 production and CD4+ T cell depletion did not differ significantly between the wt and Y86F SIVcol Nef constructs (Fig 10A–10C). Thus, SIVcol Nef modestly increased viral replication and cytopathicity in HLT, independently of its anti-SERINC5 activity.

The result that SERINC5 antagonism did not significantly affect HIV-1 replication in human T cells or *ex vivo* lymphoid tissues may seem surprising but is in agreement with previous data showing that the ability of Nef to down-modulate CD4 rather than to enhance virion infectivity correlates with its potency in stimulating viral replication in primary T cells [51,52]. SIVcol Nef is unable to down-modulate CD4 from the cell surface and promoted HIV-1 replication only in *ex vivo* HLT, but not in prestimulated CD4+ T cells. Thus, we examined whether SIVcol Nef might modulate the activity of transcription factors known to play key roles in viral gene expression. Indeed, SIVcol Nef significantly increased the activity of NF-κB (by about 30%), although this enhancing effect was weaker than the one observed for other primate lentiviral Nef proteins (Fig 10D) [19]. Similarly, both wt and Y86F SIVcol Nefs promoted the activity of NF-AT, albeit less effectively than the HIV-1 NA7 Nef (Fig 10E). These results suggest that the SIVcol Nef might be able to moderately increase HIV-1 replication in *ex vivo* HLT by enhancing NF-κB and NF-AT activity.

## Discussion

In the present study, we show that SIVcol Nef proteins derived from naturally infected mantled guerezas lack the ability to down-modulate CD4, CD28 and CD3 but are highly potent in modulating CXCR4, suppressing TCR-induced actin remodeling, impairing T cell chemotaxis towards SDF-1α, counteracting *Colobus* tetherin, and enhancing viral infectivity by counteracting SERINC3 and 5. These results expand previous studies [10,20,28] suggesting that Nef proteins from SIVcol are functionally distinct from all other known primate lentiviral Nef proteins. SERINC5 counteraction by SIVcol Nef was highly efficient and involved surface down-modulation as well as effective proteasomal degradation of this restriction factor. In addition, our results show that SIVcol Nefs are broad-spectrum antagonists of vertebrate SERINC5 proteins and fully active against orthologs from humans, zebrafish and frogs. These effects were disrupted by a single point mutation of Y86F allowing us to analyze the relevance of potent anti-SERINC5 antagonism by Nef for viral replication and cytopathicity. Although the conservation and apparent independent evolution of this Nef function suggest a relevant role *in vivo*, efficient SERINC5 counteraction did not significantly enhance viral spread and replication in preactivated human CD4+ T cells or in tonsillar lymphoid tissue cultures in the absence of endogenous stimulation. More pronounced effects of SERINC5 antagonism on infectious virus yield and virion infectivity were observed when human PBMC cultures were first HIV-1 infected and then PHA-activated six days later probably because activation reduces expression of this restriction factor [49].

It has been reported that knockdown of SERINC3 and SERINC5 in the human JTAg T cell line increases the infectivity of progeny *nef*-deficient HIV-1 virions by two orders of magnitude and that these restriction factors are highly expressed in primary human HIV-1 target cells [9]. Thus, our finding that potent antagonism of SERINC3/5 by SIVcol Nef did not enhance viral replication in activated CD4+ T cells and *ex vivo* infected tonsillar tissue may seem surprising. However, this does not contradict the data of Usami and colleagues [9] who examined the effects of SERINC3/5 in human macrophages that presumably contribute only little to the efficiency of viral replication in tonsillar tissues. Notably, potent antagonism of SERINC5 enhanced the infectivity of virions produced in pre-activated CD4+ T cells only 2- to 3-fold (Fig 8) but had up to 10-fold effects when PBMCs were stimulated several days after infection (Fig 9). Thus, the efficiency of SERINC5 restriction seems to be more efficient in primary T cells that are not fully activated. Our finding that SERINC5 antagonism did not enhance HIV-1 replication in *ex vivo* infected tonsillar tissues in the absence of exogenous stimulation (Fig 10) may seem unexpected. Possible reasons for this are that strongly activated CD4+ T cells might be the main producers of HIV-1 in this experimental system and/or that SERINC5 antagonism is less critical in lymphoid tissues where CD4+ T cells are densely packed and the virus may mainly spread via direct cell-to-cell transfer. Our results are in agreement with previous data showing that the ability of Nef to promote viral replication in cultured human cells and tissues correlates with the efficiency of Nef-mediated down-modulation of CD4 rather than enhancement of virion infectivity [51–53]. The fact that SIVcol Nef entirely lacks the CD4 down-modulation activity that is otherwise highly conserved among primate lentiviral Nef proteins thus provides a plausible explanation for its inability to promote viral spread and replication in prestimulated primary CD4+ T cells.

Our results suggest that the effects of Nef on HIV-1 replication in human lymphoid tissues, that do not require exogenous stimulation to allow productive viral replication, are more complex. It is well established that Nef modulates the responsiveness of virally infected T cells to stimulation and the activity of transcription factors such as NF-κB and NF-AT [19,54,55]. We found that SIVcol Nef moderately increases the activity of NF-κB in transiently transfected HEK293T cells and of NF-AT in HIV-1 infected Jurkat T cells. While further studies in primary human tissues are required to obtain more definitive proof, stimulatory effects on the activity of these transcription factors are a plausible explanation for the ability of SIVcol Nef to moderately enhance viral replication of HIV-1 in human tissues independently of SERINC3/5 antagonism and CD4 down-modulation.

Primate lentiviral Nef proteins show high sequence diversity. Some functional motifs, however, such as the N-terminal myristoylation site required for Nef’s association with cellular membranes, an acidic domain involved in MHC-I down-modulation, a proline-rich motif that mediates interactions with signaling molecules and an ExxxLL motif critical for interaction of Nef with adaptor protein (AP) complexes and sorting into clathrin-coated pits are usually conserved [56]. Of these, only the N-myristoylation site and the acidic cluster are preserved in SIVcol Nefs. It has been shown that the ExxxLL AP2 interaction site is critical for Nef’s ability to down-modulate CD4 and CD28 [25,30], as well as SERINC5 antagonism [8,10] but dispensable for down-modulation of MHC-I and TCR-CD3 [57–59]. In SIVcol Nefs, this motif is generally altered to YxxxLL and mutation to YxxxAA did not significantly affect its anti-SERINC5 activity [10]. Although the lack of the CD4, CD28 and Ii modulation functions suggests that SIVcol Nefs might lack a functional AP interaction site in their C-terminal loop region, mutation of YxxxLL to YxxxAA disrupted the CXCR4 down-modulation function (Fig 4B). Potentially, phosphorylation of Y204 in the YxxxLL motif, as previously reported for serine or threonine residues [60], might generate a functional AP interaction site. Notably, all SIVcol Nefs contain an aspartic acid (D) adjacent to the Y residue constituting a DxxLL motif that might potentially interact with GGA adaptor proteins [61]. Thus, the role of the YDxxLL motif in SIVcol Nef warrants further investigation. It also remains to be fully elucidated how Y86 allows SIVcol Nefs to counteract various SERINC proteins with high potency. Reduced signal intensity in mammalian-membrane two-hybrid assay (Fig 8B) suggests that Y86 might be directly involved in the interaction of SIVcol Nef with its SERINC target.

Nef proteins from SIVcol strongly down-modulated CXCR4. It has been shown that Nef-mediated down-modulation of this entry cofactor might help some primate lentiviruses to prevent superinfection [28,62]. However, the efficiency of Nef-mediated down-modulation of CXCR4 does not correlate with the coreceptor tropism of primate lentiviruses, e.g. the Nef proteins of CXCR4-tropic HIV-1 strains are poorly effective while several SIVs that use CCR5 and alternative coreceptors but not CXCR4 down-modulate this receptor with high efficiency from the cell surface [14]. It has been suggested that these primate lentiviruses might down-modulate CXCR4 to inhibit migration of T cells towards the SDF-1 chemokine and hence to impair proper lymphocyte trafficking thought to play an important role in the antiviral immune response [14]. In fact, previous studies suggest that primate lentiviral Nef proteins target cellular trafficking by several distinct mechanisms [11,12,63]. Our finding that CXCR4 down-modulation and suppression of T cell migration are conserved functions of SIVcol Nefs that lack several otherwise conserved activities supports an important role of these activities for viral replication *in vivo*. For example, effects of Nef-mediated down-modulation of CXCR4 on T cell trafficking to lymphoid tissues might clearly attenuate the efficiency of the antiviral immune response without exerting direct effects on viral replication.

The results of the present study add to the emerging evidence that SIVcol is not only evolutionarily isolated but also functionally distinct from other primate lentiviral lineages. Lack of some Nef activities might help explaining why the reported prevalence of SIVcol in the wild (on average 22.6%) is lower than that of other SIVs encoding Nef proteins that are potent SERINC5 antagonists [10]. However, the possibility that SIVcol exerts some activities in a species-specific manner or that some functions are exerted by distinct mechanisms cannot be dismissed. For example, it has recently been shown that SIVcol uses Vpr to suppress NF-κB-mediated immune activation while HIV-1 uses Vpu and many other primate lentiviruses Nef-mediated down-modulation of CD3 to achieve this [20]. Furthermore, HIV-1 uses three of its proteins (Env, Vpu and Nef) to down-modulate CD4 cell surface expression [64] and it will be of interest to examine whether SIVcol, which lacks a *vpu* gene and encodes a Nef that is inactive against CD4, has evolved alternative mechanisms to counteract inhibitory effects of this receptor. Similarly, some primary HIV-1 strains not only use Nef to counteract SERINC5 but also express Env proteins capable of evading this restriction factor [65,66]. It is not known whether SIV Envs may also confer resistance against SERINCs. Notably, only Nef but not Env prevents incorporation of SERINC5 into HIV-1 particles thereby reducing viral susceptibility to neutralizing antibodies and inhibitory peptides [65,66]. Finally, it is interesting that the highly divergent C-terminus of SIVcol Nef contains a putative MAP kinase docking domain [35] that might compensate for the lack of the proline-rich (PxxP) motif that mediates interaction with the SH3 domains of various kinases in the case of HIV-1 Nef proteins [36]. Altogether, our current knowledge suggests that SIVcol has evolved mechanisms different from other HIV and SIV strains to evade antiviral immunity and manipulate its host cell for efficient replication.

It is conceivable that increased virion infectivity should provide a selection advantage for the virus *in vivo*. However, the contribution of this specific Nef function to virus spread and replication remains to be determined. Previous studies in the SIVmac/macaque model are in agreement with a significant contribution of Nef-mediated infectivity enhancement to viral replication *in vivo* but come with the caveat that other Nef functions, such as CD4 down-modulation, were also affected [15]. The efficiency of Nef-mediated SERINC5 antagonism did not significantly affect HIV-1 replication in pre-activated CD4+ T cells and in *ex vivo* infected lymphoid tissues but correlates with the reported prevalence of SIV in their natural non-human primate hosts in the wild [10]. Thus, it is tempting to speculate that SERINC5 counteraction is more important for virus transmission than for replication within an infected individual. One plausible explanation would be that yet-to-be-identified cells expressing relatively high levels of SERINC5, such as incompletely activated CD4+ T cells or macrophages, might shed virus particles into genital fluids. In addition, SERINC5 antagonism is most likely important for viral evasion of the humoral immune response since virion incorporation of SERINC5 increases sensitivity to neutralizing antibodies [65,66]. Notably, the wild-type and Y86F SIVcol Nefs represent useful tools to examine the relevance of SERINC5 counteraction for viral replication *in vivo*, e.g. in humanized mouse or rhesus macaque model.

## Materials and methods

### Ethical statement

Experiments involving human blood, CD4+ T cells or tonsillar tissue, were reviewed and approved by the Institutional Review Board (i.e. the Ethics Committee of Ulm University). Individuals and/or their legal guardians provided written informed consent prior to donating blood or tonsillar tissues. All human-derived samples were anonymized before use. The use of established cell lines (HEK293T, TZM-bl and Jurkat cells) did not require the approval of the Institutional Review Board.

### Proviral constructs

Proviral HIV-1 NL4-3-IRES-eGFP constructs containing an internal ribosome entry site (IRES) and the gene encoding the enhanced version of the green fluorescent protein (eGFP) were generated as described [32]. Overlap-extension PCR was used to replace the NL4-3 *nef* in wt or IRES-eGFP HIV-1 constructs by different primate lentiviral *nef* genes as described [6]. The integrity of all PCR-derived inserts was confirmed by sequencing. The control HIV-1 NL4-3-IRES- eGFP constructs expressing the NL4-3, NA7, and SIVmac239 Nefs or containing a disrupted *nef* gene have been reported previously [7].

### Expression vectors

Cloning of *nef* alleles into the bi-cistronic CMV promoter-based pCG expression vector co-expressing the enhanced green fluorescent protein (eGFP) or blue fluorescent protein (BFP) was described previously [6,67]. In brief, *nef* genes were amplified by PCR with flanking primers introducing XbaI and MluI restriction sites for cloning into the pCG IRES eGFP or BFP vectors. Mutant Nef variants (SIVcol Y86F, Y87F Nef) were generated by mutating TAT (Tyr86) to TTC (Phe) and TAC (Tyr87) to TTC (Phe). Sequence of tetherin from *Colobus guereza kikuyuensis* was obtained from the public database and synthesized. Internal Mlul restriction site was removed without changing the protein amino acid sequence (GCG to GCC). *Colobus* tetherin was cloned into pCG expression vector co-expressing BFP as described [60] and confirmed by sequence analysis. Human tetherin pCG expression vector was used previously [17]. MaMTH Nef N-Bait and SERINC5 C-Prey vectors were generated using *Att* recombination site-based Gateway cloning technology (Invitrogen). The original Prey and Bait expression vectors have been described [43,68], and all expression constructs were confirmed by sequence analysis. The pBJ6 vector expressing HA-tagged SERINC5, pBJ5 vector expressing HA-tagged MLV glycoGag as well as the pSERINC-GFP vectors have been previously reported [8]. Human SERINC5 mutants L350A and I352A, as well as frog and zebrafish orthologs and frog/human SERINC5 chimeras were described before [39].

### Cell culture and transfection

Human Embryonic Kidney (HEK) 293T cells (obtained from the American Type Culture Collection (ATCC)) were first described by [69]. TZM-bl reporter cells (kindly provided by Drs. Kappes and Wu and Tranzyme Inc. through the NIH AIDS Reagent Program [70] were used to determine infectious virus yield. Both cell lines were cultured in Dulbecco’s Modified Eagle Medium (DMEM) supplemented with 10% heat-inactivated fetal calf serum (FCS), 2 mM L-glutamine, 100 units/ml penicillin and 100 μg/ml streptomycin. TZM-bl cells express CD4, CCR5 and CXCR4 and contain the β-galactosidase genes under the control of the HIV-1 promoter [70,71]. HEK293T B0166 *Gaussia* luciferase reporter cells [43] maintained in Dulbecco’s Modified Eagle Medium (DMEM) supplemented with 10% heat-inactivated fetal calf serum (FCS) and antibiotics. Jurkat T (obtained from the American Type Culture Collection (ATCC)) were generated by Schneider et al. [72] and cultured as described previously [73,74]. Jurkat T-cell clones expressing an HA-coding sequence in exon 8 of *serinc5* alleles were generated using CRISPR/Cas9-assisted gene editing, resulting in the expression of endogenous SERINC5 protein displaying an HA epitope in its predicted fourth extracellular loop at the interface of E290/H291.

Peripheral blood mononuclear cells (PBMCs) from healthy human donors were isolated using lymphocyte separation medium (Biocoll separating solution; Biochrom), stimulated for 3 days with phytohemagglutinin (PHA; 2 μg/ml), and cultured in RPMI 1640 medium with 10% fetal calf serum and 10 ng/ml interleukin-2 (IL-2) prior to infection (dx.doi.org/10.17504/protocols.io.r7cd9iw) or first infected and then cultured in medium only until stimulation with PHA at day 3 or 6. CD4+ T cells from healthy human donors were isolated using RosetteSep Human CD4+ T Cell Enrichment Cocktail (Stemcell) and lymphocyte separation medium (Biocoll separating solution; Biochrom), stimulated for 3 days with phytohemagglutinin (2 μg/ml), and cultured in RPMI 1640 medium with 10% fetal calf serum and 10 ng/ml interleukin-2 (IL-2) prior to infection (dx.doi.org/10.17504/protocols.io.r7ad9ie). Transfection of Jurkat T cells was performed using the DMRIE-C reagent (GibcoBRL) following the manufacturer's instructions.

### Western blot

To examine the expression of primate lentiviral Nef proteins, HEK293T cells were transfected in 6-well dishes with 5 μg DNA of pCG IRES eGFP vectors expressing AU1-tagged Nefs. Two days post-transfection, cells were lysed with Co-IP buffer (150 mM NaCl, 50 mM HEPES, 5 mM EDTA, 0.10% NP40, 0.5 mM sodium orthovanadate, 0.5 mM NaF, protease inhibitor cocktail from Roche) and reduced in the presence of β-mercaptoethanol by boiling at 95°C for 10 min. Proteins were separated in 4 to 12% Bis-Tris gradient acrylamide gels (Invitrogen), blotted onto polyvinylidene difluoride (PVDF) membrane, and incubated with anti-AU1 (MMS-130P; Covance), anti-GFP (ab290; Abcam), and anti-β-actin (ab8227; Abcam) antibodies. Subsequently, blots were probed with anti-mouse and anti-rabbit IRDye Odyssey antibodies (cat.no. 926–32210 and 926–32221; LI-COR), and scanned using a LI-COR Odyssey reader. Western blot analysis of cell-associated and virion levels of SERINC5 and HIV-1 Gag was conducted as previously described [75].

### Flow cytometry

CD4, MHC-I, CD28, CD3 and GFP reporter molecules in PBMCs and CXCR4 surface levels in Jurkat T cells transduced with HIV-1 NL4-3 constructs coexpressing Nef and GFP were measured as described previously [73]. Up-modulation of Ii cell surface expression was measured on THP-1 cells. The following phycoerythrin-conjugated antibodies were used: anti-human CD4, anti-human CD3 and anti-LeuTM-28 (BD Biosciences), anti-CD74/R-PE M-B741 (Ancell), anti-HLA-ABC Antigen/RPE (DAKO), mouse anti-human HLA-DR TUE36 (Caltag laboratories) and L243 (BD Biosciences). Nef-mediated down- or up-regulation of cellular receptors was quantitated as described [6]. To determine CXCR4 modulation by Nef, pre-stimulated human PBMCs were transduced with NL4-3-based IRES-eGFP viral constructs. Surface expressed CXCR4 was stained 3 days post-transduction using CXCR4 APC (BD, cat. no. 555976) conjugated antibody. To determine total cellular CXCR4 levels, transduced PBMCs were permeabilized using Fix&Perm kit (MUBio, cat.no. GAS-002-1) prior to CXCR4 staining. CXCR4 down-modulation was calculated by dividing the mean fluorescence intensity (MFI) values obtained for cells transduced with viruses co-expressing Nef by the MFI of cells transduced with the *nef*-defective control construct. To determine SERINC5 surface downmodulation and degradation, Jurkat cells engineered to express endogenous SERINC5 with HA tag in the predicted fourth extracellular loop were transduced with VSV-G pseudotyped NL4-3 IRES eGFP viruses encoding indicated *nefs* or mutants thereof. After 40h, cells were treated for 6h with 10 μM MG132 proteasomal inhibitor, 10 mM NH_4_Cl or medium only (untreated). To measure total cellular SERINC5 levels, the cells were permeabilized using FIX&PERM Cell Fixation and Permeabilization Kit and stained with anti-HA antibody conjugated to Alexa Fluor 647 (BioLegend #682404). To measure surface SERINC5 levels, unpermeabilized cells were stained in the same manner before fixation with 2% PFA. As a negative staining control, cells were treated with Alexa Fluor 647 Mouse IgG1 Isotype Control antibody. Levels of Alexa Fluor 647 and eGFP were determined by flow cytometry.

### Virus stocks

To generate virus stocks, HEK293T cells were co-transfected with the proviral HIV-1 NL4-3 constructs encoding containing various *nef* alleles and a plasmid (pHIT-G) expressing VSV-G to achieve comparably high infection levels for flow cytometric analysis and replication kinetics. Two days post-transfection, supernatants containing infectious virus were harvested. The amount of HIV-1 capsid protein was quantified by p24 antigen ELISA as described [76] for normalization of the virus dose.

### Viral infectivity

Virus infectivity was determined using P4-CCR5 and TZM-bl cells as described previously [7]. Briefly, the cells were sown out in 96-well dishes in a volume of 100 μl and infected by overnight incubation with virus stocks, containing 1 ng of p24 antigen, produced by transfected HEK293T cells. Three days post-infection, viral infectivity was detected using the Gal-Screen kit from Applied Biosystems as recommended by the manufacturer. β-galactosidase activity was quantified as relative light units per second using the Orion microplate luminometer.

### SERINC antagonism

To measure Nef-mediated SERINC3 and SERINC5 counteraction, HEK293T cells were co-transfected using calcium phosphate with 3 μg of HIV-1 NL4-3 IRES GFP reporter proviral constructs containing various *nef* alleles and 2.5 μg pBJ6-SERINC5-HA expression plasmid or pBJ6-empty vector (6-well format). The HIV-1 NL4-3 *nef*- construct was used as negative control and viral particles produced by co-transfection with 500 ng HA-tagged MLV glycoGag served as positive control. To measure Nef-mediated counteraction of SERINC5 mutants, chimeras and orthologs, HEK293T cells were co-transfected using polyethylenimine (PEI) with 0.9μg HIV-1 NL4-3 IRES GFP reporter proviral constructs containing various *nef* alleles and 0.1μg pBJ5 SERINC (24-well format). The HIV-1 NL4-3 *nef*- construct was used as negative control and viral particles produced by co-transfection with 0.1μg HA-tagged MLV glycoGag or pHIT-G vector expressing VSV-G served as positive controls. Two days post-transfection, cell supernatants were harvested and infectious HIV-1 yield was quantified by TZM-bl infection assay [7].

### Tetherin antagonism

To measure SIV-col Nef-mediated human and *Colobus* tetherin counteraction, HEK293T cells were co-transfected using calcium phosphate method with 4 μg HIV-1 NL4-3 provirus lacking Nef and Vpu expression (Δ*nef*Δ*vpu*), 1 μg Nef expression vector or empty vector as well as increasing amounts of human or *Colobus* tetherin DNA (0–0.25 μg). Two days post-transfection, supernatants and cells were harvested. Infectious HIV-1 yield was quantified by a 96-well TZM-bl infection assay whereas p24 concentration was determined by p24 ELISA quantification of cell free (CF) and cell associated (CA) capsid p24 antigen. Release was calculated as the percentage of CF p24 out of total (CF + CA) produced p24 antigen.

### T cell migration

Chemotaxis of Jurkat T (CCR7) cells towards SDF-1α (10 ng/ml) was determined in a transwell system (5 μm pore size) for 2 h after starvation in hunger medium (0.5% FCS). Percentages of migrated cells were calculated from % GFP expressing cells after migration relative to % GFP expressing cells given as input per condition.

### Lck retargeting

Jurkat-TAg T cells transiently expressing Nef or control cells were plated onto coverslips, fixed, permeabilized and stained for endogenous Lck essentially as described previously [77,78].

### Actin ring formation

Effects of Nef on the actin skeleton were analyzed essentially as described previously [77,79]. Briefly, microscope cover glasses were coated with anti-CD3 antibody diluted in TBS (10 μg/ml) for 3 h at 37°C. After being washed with TBS, the cover glasses were stored in TBS at 4°C. Cells were added in a volume of 50 μl onto the glasses, incubated for 5 min at 37°C, fixed by direct addition of paraformaldehyde and subsequently analyzed by widefield microscopy.

### Confocal microscopy

JTAg SERINC3/5 KO cells (a gift from M. Pizzato) were electroporated in 0.4 cm gap cuvettes (250 V, 950 μF in PBS containing 1% DMSO) with 15 μg p_SERINC5-GFP and 15 μg pCG-Nef-AU1 expression plasmids, using Xcell Gene pulser (Bio-Rad) and incubated for 40h. Cells that were later to be stained with proteasomal markers were treated with 10 μM MG132 for 3h or left untreated prior to harvesting. Cells were washed, fixed in 2% PFA, permeabilized and blocked using 5% BSA, 0.2% TritonX in DPBS. Nef was stained using mouse monoclonal AU1 (MMS130R, Covance, 1:500), followed by anti-mouse APC (Invitrogen, A-865, 1:1000). Lysosomes were stained with rabbit anti-LAMP (abcam, ab24170, 1:500) and proteasome was stained with rabbit anti-PSMA5 (ThermoFisher, PA5-17295, 1:250) followed by anti-rabbit AlexaFluor568 (Invitrogen, A10042, 1:1000). Hoechst stain was used to detect nuclei. Cells were mounted in Glycerol Mounting Medium in IBIDI microslides. Laser scanning microscopy was performed using a Zeiss LSM 710.

### Image processing and analysis

Raw image stacks were deconvoluted using a calculated point spread function in Huygens Professional (Scientific Volume Imaging) and further analyzed and displayed using both ImageJ and Huygens Professional. Pearson correlation coefficient per individual cell was calculated using Huygens Professional (Scientific Volume Imaging) with Costes automatic thresholding. Pixel area of immunofluorescence staining was determined in ImageJ by using automatic thresholding and quantifying the extracted stained pixels per individual cell.

### Viral replication kinetics in CD4+ T cells

CD4+ T cells pre-stimulated with phytohemagglutinin (2 μg/ml) were transduced with VSV-G pseudotyped virus stocks containing normalized quantities of p24 antigen. Four hours post-transduction, cells were washed three times in PBS, split into multiple wells of a 96-well plate for each used donor (5 x 10^5^ cells/well) and resuspended in RPMI 1640 medium with 10% heat-inactivated FCS, 2 mM L-glutamine and antibiotics. Transduced cells were cultured with or without agitation (650 rpm) for up to 10 days. Every second day, 80% of the culture supernatant was harvested for determination of infectious virus and p24 yield by TZM-bl infection or p24 antigen ELISA, respectively. In addition, the percentage of HIV-1 infected p24+ T cells was determined by intracellular staining using Fix&Perm kit (MUBio, cat.no. GAS-002-1) and a FITC-conjugated p24 antibody (Beckman Coulter, clone KC57) followed by flow cytometric analysis.

### Viral replication kinetics in PBMCs

A total of 7 x 10^5^ PBMCs/well were transduced in triplicates with VSV-G pseudotyped virus stocks containing normalized quantities of p24 antigen. Six hours post-transduction, cells were washed with PBS and resuspended in RPMI 1640 medium containing 10% heat-inactivated FCS, 2 mM L-glutamine and antibiotics. Cells were stimulated with 2 μg/ml phytohemagglutinin and 10 ng/ml IL-2 either 3 days prior to transduction, 3 days after transduction, or 6 days after transduction and maintained in RPMI 1640 medium with 10% heat-inactivated FCS, 10 ng/ml IL-2, 2 mM L-glutamine and antibiotics until day 15. Every third day, 80% of the culture supernatant was harvested for determination of infectious virus yield by TZM-bl infectivity assay as well as reverse transcriptase activity by radioactivity-based RT assay described before [7].

### qRT-PCR

PBMCs were obtained from the same donors as used in the PBMC kinetic described above. Cells were cultured for 3 days in RPMI 1640 medium with 10% fetal calf serum and antibiotics, containing either phytohemagglutinin (2 μg/ml), 10 ng/ml interleukin-2 (IL-2) or both. RNA was isolated using RNeasy Plus Mini Kit (Qiagen 74136) and reverse transcribed with Prime Script RT reagent kit (Takara #RR037A). Gene expression was measured using TaqMan Fast Universal PCR Master Mix (ThermoFisher #4352042) and the following TaqMan probes: SERINC5 (ThermoFisher #Hs00968169_m1) and as a normalization control, GAPDH (ThermoFisher #Hs99999905_m1). Ct data was processed relative to the GAPDH control and further normalized to control cells cultured in the absence of IL-2 and PHA from the same donor.

### SERINC5 and Nef interaction assay

The Mammalian Membrane Two-Hybrid (*MaMTH*) Assay was performed as described [43,68]. In brief, HEK293T B0166 *Gaussia* luciferase reporter cells were co-transfected in 96-well plates with MaMTH vectors including 25 ng Nef Bait and 25 ng SERINC5 Prey DNA in triplicates using PEI transfection reagent. Gal4 (transcription factor) as well as EGFR Bait with SHC1 Prey served as positive controls, whereas Nef Bait proteins with Pex7 Prey were used as negative controls. After 24 h, Bait protein expression was induced by adding 0.5 μg/ml tetracycline. Supernatants were harvested 40 h post-transfection and the released *Gaussia* reporter was measured 1 s after injecting 20 mM coelenterazine substrate using a BMG Omega Plate luminometer.

### NF-AT assay

Jurkat cells stably transfected with an NF-AT-dependent reporter gene vector [54] were either left uninfected or transduced with HIV-1 IRES eGFP constructs expressing various *nef* alleles. Except for those cells used as controls, cultures were treated with PHA (1 μg/ml; Murex). Luciferase activity was measured and n-fold induction determined by calculating the ratio of measured relative light units (RLU) of treated samples to that of untreated samples, as described previously [80].

### NF-κB reporter assay

To determine the effect of Nef on NF-κB activity, HEK293T cells were seeded in 96-well plates coated with poly-L-lysine and cotransfected with a firefly luciferase reporter construct under the control of three NF-κB binding sites, a *Gaussia* luciferase construct under the control of a constitutively active pTAL promoter for normalization, and expression vectors for different Nef proteins as described [20]. Two days post-transfection, a dual luciferase assay was performed and the firefly luciferase signals were normalized to the corresponding *Gaussia* luciferase control values.

### *Ex vivo* human lymphoid tissue

Human tonsillar tissue removed during routine tonsillectomy and not required for clinical purposes was received within 5 h of excision. The tonsils were washed thoroughly with medium containing antibiotics and then sectioned into 2 to 3 mm^3^ blocks. These tissue blocks were placed on top of collagen sponge gels in the culture medium at the air-liquid interface and infected without exogenous stimulation as described [51]. Supernatants were collected at 3 day intervals, and productive HIV-1 infection was assessed by measuring p24 antigen content. Flow cytometry was performed on cells mechanically isolated from control and infected tissue blocks, and depletion of CD4+ T cells was quantified as described previously [81].

### Phylogenetic analysis

Sequences were derived from the Los Alamos sequence database (http://hiv-web.lanl.gov). Phylogenetic tree construction was done with the Phylogenetic analysis tool: “Phylogeny.fr” [82].

### Statistical methods

The mean activities were compared using Student’s t-test. Similar results were obtained with the Mann Whitney test. The software package StatView version 4.0 (Abacus Concepts, Berkeley, CA) was used for all calculations.

## Supporting information

S1 Movie Localization of SERINC5. Jurkat SERINC3/5 K.O. cells were transfected with SERINC5-GFP (green). Nuclei were stained with Hoechst (white). Z-stacks were taken using Zeiss LSM710 laser scanning microscope, images were deconvoluted using Huygens Professional and 3D movies rendered in Imaris.
(MP4)

S2 Movie Localization of SERINC5 in the presence of HIV-1 Nef. Jurkat SERINC3/5 K.O. cells were transfected with SERINC5-GFP (green) and HIV-1 Na7 Nef AU1. Cells were stained for AU1 (red) and endogenous LAMP-1 (blue). Z-stacks were taken and movies generated as described in the legend to S1 Movie.
(M4V)

S3 Movie Localization of SERINC5 in the presence of SIVcol Nef. Jurkat SERINC3/5 K.O. cells were transfected with SERINC5-GFP (green) and SIVcol Nef AU1. Cells were treated with 10 μM of MG132 proteasomal inhibitor to visualize co-localization. Samples were stained for AU1 (red), endogenous PSMA5 (blue) and nucleus (Hoechst; white). Z-stacks were taken and movies generated as described in the legend to S1 Movie.
(M4V)

S4 Movie Localization of SERINC5 in the presence of SIVcol Y86F mutant Nef. Jurkat SERINC3/5 K.O. cells were transfected with SERINC5-GFP (green) and SIVcol Y86F Nef AU1. Cells were treated with 10 μM of MG132 proteasomal inhibitor to visualize co-localization. Samples were stained for AU1 (red), endogenous PSMA5 (blue) and nucleus (Hoechst; white Z-stacks were taken and movies generated as described in the legend to S1 Movie.
(M4V)
